# The Spliceosomal Phosphopeptide P140 Controls the Lupus Disease by Interacting with the HSC70 Protein and via a Mechanism Mediated by γδ T Cells

**DOI:** 10.1371/journal.pone.0005273

**Published:** 2009-04-23

**Authors:** Nicolas Page, Nicolas Schall, Jean-Marc Strub, Marc Quinternet, Olivier Chaloin, Marion Décossas, Manh Thong Cung, Alain Van Dorsselaer, Jean-Paul Briand, Sylviane Muller

**Affiliations:** 1 CNRS UPR9021, Institut de biologie moléculaire et cellulaire, Strasbourg, France; 2 CNRS UMR7178, laboratoire de spectrométrie de masse BioOrganique-IPHC-DSA- Université de Strasbourg, Strasbourg, France; 3 CNRS-INPL UMR7568, Laboratoire de Chimie-Physique Macromoléculaire, Nancy Université, ENSIC, Nancy, France; Centre de Recherche Public de la Santé (CRP-Santé), Luxembourg

## Abstract

The phosphopeptide P140 issued from the spliceosomal U1-70K snRNP protein is recognized by lupus CD4^+^ T cells, transiently abolishes T cell reactivity to other spliceosomal peptides in P140-treated MRL/lpr mice, and ameliorates their clinical features. P140 modulates lupus patients' T cell response ex vivo and is currently included in phase IIb clinical trials. Its underlying mechanism of action remains elusive. Here we show that P140 peptide binds a unique cell-surface receptor, the constitutively-expressed chaperone HSC70 protein, known as a presenting-protein. P140 induces apoptosis of activated MRL/lpr CD4^+^ T cells. In P140-treated mice, it increases peripheral blood lymphocyte apoptosis and decreases B cell, activated T cell, and CD4^−^CD8^−^B220^+^ T cell counts via a specific mechanism strictly depending on γδ T cells. Expression of inflammation-linked genes is rapidly regulated in CD4^+^ T cells. This work led us to identify a powerful pathway taken by a newly-designed therapeutic peptide to immunomodulate lupus autoimmunity.

## Introduction

The U1-70K small nuclear ribonucleoparticle protein is a major spliceosomal autoantigen recognized in systemic lupus erythematosus (SLE). We previously identified an epitope between residues 131–151 present within its RNA recognition motif and targeted early during the progression of the disease by IgG antibodies and CD4^+^ lymph node cells (LNCs) from H-2^k^ MRL/lpr and H-2^d/z^ (NZBxNZW)F1 lupus-prone mice [1], [2]. A peptide analogue phosphorylated on Ser^140^ (named P140) was also recognized by LN and peripheral MRL/lpr CD4^+^ T cells [3]. Intravenous administration into Fas(CD95)-deficient MRL/lpr mice of P140 significantly improved their clinical and biological manifestations and prolonged their survival, while the non-phosphorylated analogue did not [3]. Moreover, when incubated with lupus patient's peripheral blood lymphocytes (PBLs), P140 generated secretion of high levels of regulatory cytokine IL-10 in cell cultures without proliferation of CD4^+^ T cells, suggesting that P140 (and not the non-phosphorylated analogue, which induces CD4^+^ T cell proliferation) possesses specific immunomodulatory functions on lupus T cells [4]. This assumption was supported by showing that repeated administration of P140 into MRL/lpr mice transiently abolishes T cell reactivity to other regions of the U1-70K protein and to epitopes from other spliceosomal proteins [5], [6] without altering the capacity of P140-treated mice to mount a normal protective antiviral immune response [6]. P140 was successfully included in a phase IIa clinical trial [7], and is currently evaluated in a phase IIb, double-blind, placebo-controlled dose-ranging study.

The present study was performed to decipher the P140 mode of action. We sought putative receptor(s), different from the MHC molecules, which might explain the remarkable *in vivo* efficacy of P140, either alone or synergistically with class II MHC-peptide-T cell receptor (TCR) interaction. This led us to identify at the surface of spleen cells and LNCs a very specific P140-receptor, the heat-shock cognate HSC70 protein, and to further investigate whether the P140 phosphopeptide acts *via* γδ T cells. These regulatory T cells, which control αβ T cells, activated B cells and NK cells, preferentially respond to phospholigands [8] and interact with HSC70 [9], [10]. They are abnormally regulated in human and murine lupus [11], [12]. We also examined the genes that are differentially expressed rapidly after P140 administration into MRL/lpr mice. The results indicate that P140 controls the lupus disease by a unique mechanism involving pathways of both innate and acquired immune responses.

## Results

### P140 recognizes cell surface-expressed HSC70 protein

**Figure 1:**
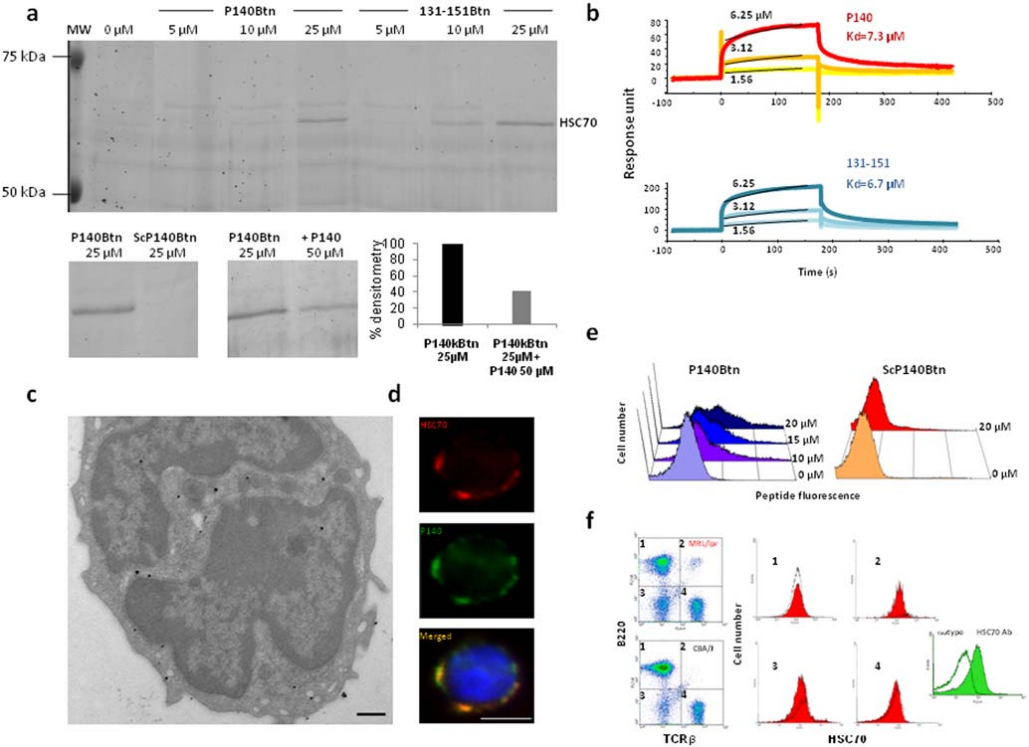
Peptide P140 binds the constitutively-expressed chaperone HSC70 protein (A) Biotin-labeled P140 and 131-151 peptides form a stable and specific, dose-dependent complex with HSC70 expressed on the surface of CBA/J and MRL/lpr splenocytes. The same results were obtained with LNCs from normal and lupus mice. There is no binding when a biotin-labeled scrambled form of P140 peptide (ScP140 Btn) is used instead of P140 peptide, and the binding is inhibited by an excess of free, non-biotinylated peptides (see the quantification by densitometry). (B) Direct binding to recombinant HSC70 protein of P140 and non-phosphorylated 131–151 peptides used at increasing concentrations (1.56 to 12.5 µM), as measured by surface plasmon resonance experiments. Analysis was performed using the simple 1∶1 Langmuir binding model, the fitting to each model was judged by the χ^2^ value and randomness of residue distribution was compared to the theoretical model. (C) Subcellular localization of HSC70 shown by immunoelectron microscopy. Fixed and permeabilized MRL/lpr PBLs were incubated overnight with the anti-HSC70 rat monoclonal antibody 1B5, followed by incubation with goat anti-rat IgG conjugated to ultra-small gold particles. Gold particles were enhanced using a silver kit. Cells were then treated for transmission electron microscopy with conventional methods (bar = 500 nm). (D) Co-localization, at the surface of non-permeabilized MRL/lpr splenocytes, of P140 peptide and HSC70 staining as shown by fluorescent microscopy: P140 followed using biotinylated peptide and FITC-labeled streptavidin, and HSC70 followed using the R-phycoerythrin-labeled anti-HSC70 rat monoclonal antibody 1B5. DAPI (4′,6′-diamidino-2-phenylindole) was used to label the nucleus (bar = 3 µm). (E) Binding in a dose-dependent manner of biotin-labeled P140 peptide (but not of ScP140 Btn) to the surface of MRL/lpr splenocytes as measured at 4°C by FACS analysis (the results obtained at 20 and 37°C are shown in Figure S4). (F) Cell-surface HSC70 expression among different CBA/J (black line) and MRL/lpr (red) cell subsets. (1) B220^+^TCRβ^−^ = B cells; (2) B220^+^TCRβ^+^ = activated T cells frequent in lupus mice, including CD4^−^CD8^−^ (DN) T cells, the number of which is raised in MRL/lpr mice; (3) B220^−^TCRβ^−^ = naive cells; (4) B220^−^TCRβ^+^ = T cells. Binding visualized with an isotypic control antibody is shown (right panel, in green).

To identify putative cell-surface receptor(s) of P140, we undertook a series of experiments based on a previously described method [13], [14] using spleen cells and LNCs from MRL/lpr mice and biotin-labeled P140. The purified proteins were subjected to SDS-PAGE in denaturing conditions and the resolved gel was stained with colloidal blue. This procedure led us to identify a single specific protein band (Figure 1A), which was identified by nano LC-MS/MS [15] as the heat-shock cognate 71-kDa protein, also termed HSC70 or Hsp/HSC73 protein (Figure S1). Twenty-six unique peptides matched between this newly characterized P140-receptor and HSC70 covered 58% of the theoretical HSC70 sequence. Identification of several discriminating peptides allowed us to clearly discard other Hsps, such as the inducible Hsp70/Hsp72. HSC70 recovered from the cell surface in these conditions was the only protein specifically bound by P140 in a dose-dependent manner (Figure 1A). HSC70 also formed a stable complex with the non-phosphorylated peptide 131–151 but not with the scrambled peptide P140 (ScP140; Figure 1A). Formation of the complex was hampered by competing P140 (Figure 1A).

To assess P140 binding to HSC70, we investigated the binding parameters and kinetics by surface plasmon resonance. HSC70 was immobilized onto the sensor chip *via* one of its four thiol groups, and different concentrations of P140 and 131–151 peptides were injected in the flux followed by a dissociation phase (see the overlay surface plasmon resonance plots of the raw data in Figure 1B). The association rate constants (k_on_) and dissociation rate constants (k_off_) were 530 M^−1^s^−1^ and 3.88×10^−3^s^−1^ for the P140 peptide, and 606 M^−1^s^−1^ and 4.07×10^−3^s^−1^ for the peptide 131–151, respectively, using Langmuir's one site model, which gave the best fit at all concentrations used (χ^2^ = 3.2/R_max_ = 75 RU and 28.0/R_max_ = 236 RU, for the P140 and 131–151 peptides, respectively). The equilibrium dissociation constant (Kd) calculated from the ratio of the kinetic rate constants (k_on_/k_off_), was 7.32 µM and 6.72 µM, respectively, for the P140 and 131–151 peptides. Similar data were obtained when biotinylated P140 was on the chip and HSC70 in solution. Thus, in this *in vitro* system, both peptides bind with medium affinity (µM range) to HSC70. ScP140 did not bind HSC70. Of significant importance, no binding was observed between P140 and Hsp70.

In good agreement with previous data showing that HSC70 is located at the plasma membrane, in the cytoplasm and nucleus [16], [17], a membrane and punctuated intracellular fluorescent labeling of anti-HSC70 antibodies was observed on permeabilized MRL/lpr (Figure S2) and CBA/J splenocytes and PBLs. By electron microscopy, HSC70 was found localized in association with the plasma and nuclear membranes, the endoplasmic reticulum, small vesicles and the nucleus (Figure 1C). After incubating non-permeabilized splenocytes of both strains with P140, HSC70 and P140-staining co-localized at the plasma membrane (Figure 1D; Figure S3).

Binding of P140 at the cell surface was confirmed by FACS analysis using freshly purified MRL/lpr splenocytes, increasing concentrations of biotin-labeled P140 and allophycocyanin-labeled streptavidin to reveal interaction. Surface P140 staining increased in a concentration-dependent manner at 4°C (Figure 1E) and 20°C (Figure S4), while very little staining was seen at 37°C, which is consistent with the internalisation of P140 associated with its HSC70 receptor. No fluorescence staining was visualized in the presence of biotin-labeled ScP140.

HSC70 staining was present at the surface of MRL/lpr and CBA/J TCRβ^+^B220^+/−^ and TCRβ^−^B220^+/−^ cells corresponding to B cells, T cells and CD4^−^CD8^−^B220^+^ double negative (DN) T cells that progressively invade MRL/lpr mice (Figure 1F). Levels of HSC70 expression at the surface of normal and MRL/lpr PBLs were similar (6-week-old mice).

### Structural and physical properties of P140 peptide

**Figure 2:**
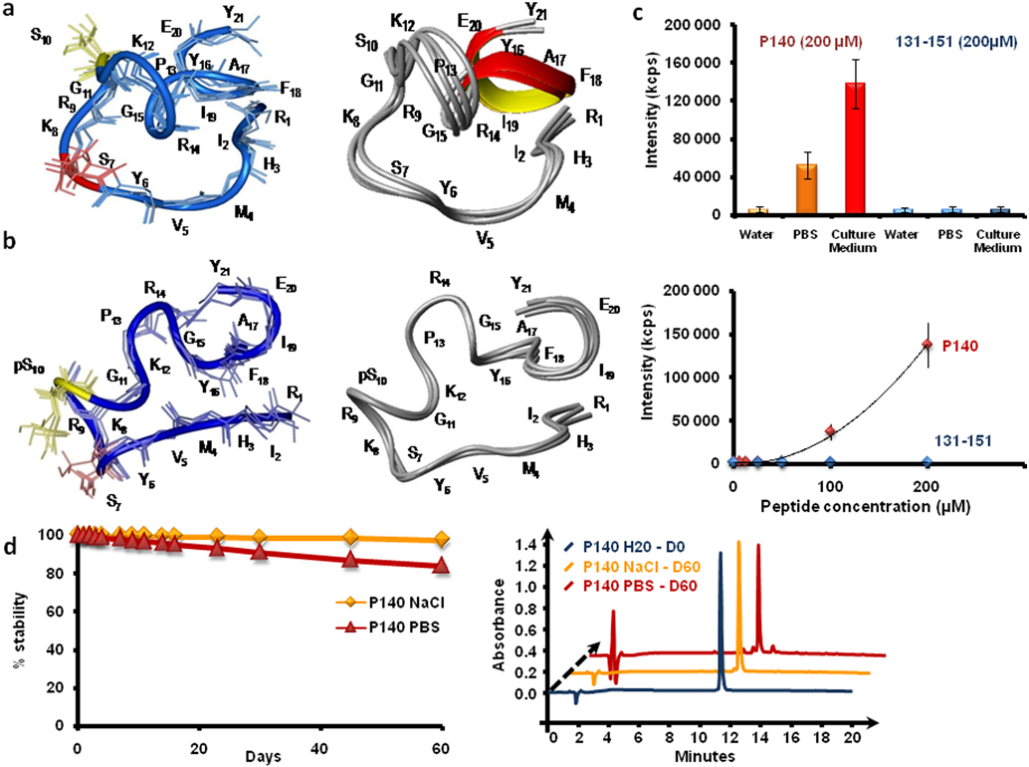
NMR structure, solubility, and stability of P140 and 131–151 peptides. (A,B) Superposition of the four lowest energy structures of the non-phosphorylated and phosphorylated peptides 131–151 after simulated annealing and restrained MD calculations. For simplicity, peptide residues are numbered from 1 to 21 for the non-phosphorylated peptide (a) and phosphorylated P140 peptide (b). The phosphorylated Ser residue at position 10 (Ser^140^) is represented by pS. For full details, see Text S1, Tables S1, S2, S3, S4, and Figures S4, S5, S6, S7. (C) Determination of the solubility limit of P140 and 131–151 peptides in distilled water, PBS and RPMI 1640 culture medium. The data show the variation of the mean intensity of light scattered (expressed as kilocounts, Kcps) that occurs when peptide aggregates are formed. In culture medium, P140 peptide aggregates at concentrations equal or superior to 50 µM. The amount and size of aggregates increase with peptide concentrations (4136±2800 nm at a 100 µM-concentration and 6972±4200 nm at a 200 µM-concentration). (D) Stability at 37°C of P140 peptide in 150 mM NaCl and PBS, as measured by high-performance liquid chromatography from the area of the peak corresponding to the intact peptide.

The above-described results show no difference regarding HSC70 recognition by the phosphorylated and non-phosphorylated peptides. Knowing that only P140 controls lupus disease in MRL/lpr mice, we evaluated conformational characteristics of both peptides by ^1^H-NMR spectroscopy and molecular dynamics calculation. The detailed determination of the peptide structures is described in the supporting information (Tables S1, S2, S3, S4, and Figures S5, S6, S7, S8). As shown in Figure 2A and 2B, the conformation of the non-phosphorylated and phosphorylated peptides appears only slightly different (see Text S1). It should be emphasized in particular that in both peptide analogues, the side-chain of the Ser^140^ and pSer^140^ residues is directed towards the exterior medium and is part of the hairpin loop, which is probably important for recognition properties.

The solubility of P140 and 131–151 peptides was determined at 20 and 37°C using dynamic light scattering measurements [18]. While a 200 µM-solution of peptide 131–151 remained highly soluble in water, phosphate-buffered saline (PBS) and culture medium (Figure 2C), aggregates form in the P140 solution at concentrations equal or superior to 50 µM in culture medium and PBS. In water, P140 was highly soluble (Figure 2C). The integrity of P140, as measured in saline and PBS by high-performance liquid chromatography from the area of the peak corresponding to the intact peptide, remained intact during at least 60 days at 37°C (Figure 2D).

### *Ex vivo*, P140 induces apoptosis of specific T cell subsets *via* a granzyme B-dependent mechanism

**Figure 3:**
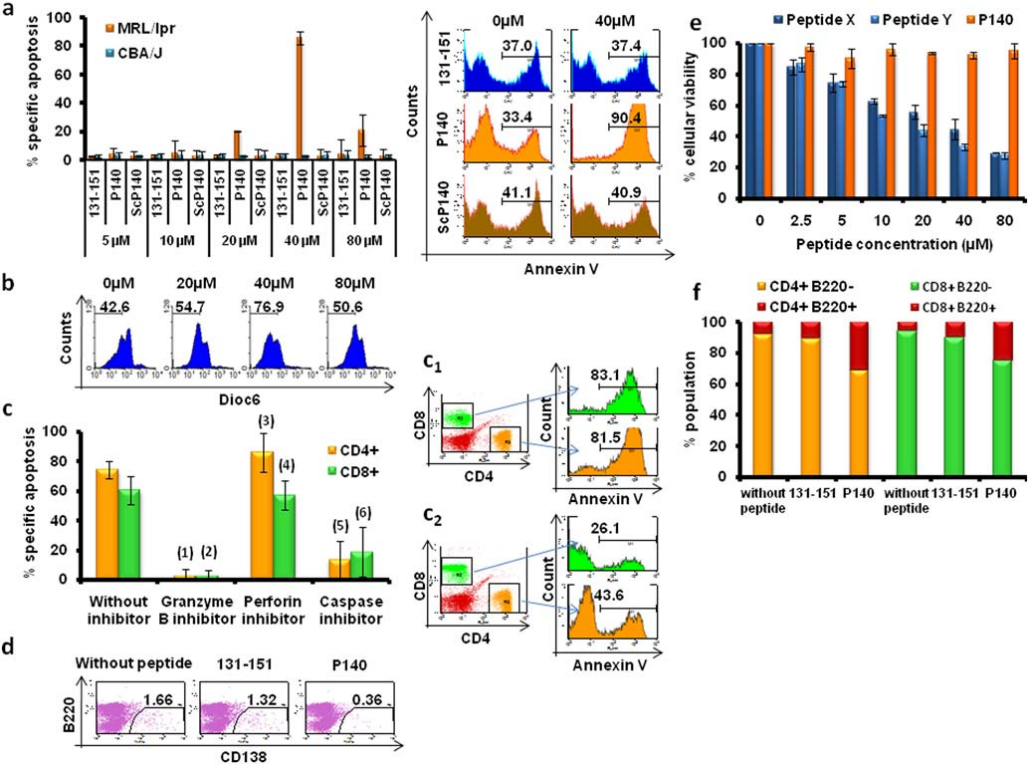
*Ex vivo* effect of P140 peptide on MRL/lpr PBL apoptosis. (A) Induction of MRL/lpr and CBA/J PBLs apoptosis as measured by flow cytometry after annexin V- allophycocyanin labeling. The effect of increasing concentrations of the three peptides P140, 131–151 and ScP140 was assayed 20h after peptide incubation with cells. The results are expressed as the mean percentage (±SD) of specific apoptosis measured in three independent experiments. The decreased effect visualized when using 80 µM P140 probably results from peptide aggregation. (B) The same experiment was performed in measuring apoptosis of MRL/lpr PBLs by the decrease of mitochondrial Δψm, as measured by a reduction of DIOC_6_ dye uptake, in the presence of increasing concentrations of P140 peptide. (C) Induction of CD4^+^ and CD8^+^ T cell apoptosis by P140 peptide and effect of granzyme-B, perforin and caspase inhibitors. Twenty hours after P140 incubation, cell death was determined by flow cytometry using an annexin V-allophycocyanin labeling (see also insert c1). Granzyme-B inhibitor I (20 µMZ-AAD-CMK; see also insert c2), perforin inhibitor (5 nM concanamycin A) and caspase inhibitors (20 µM Z-VAD-FMK) were added 30 min post-P140 incubation to analyze the involvement of these pathways. The results are expressed as the mean percentage (±SD) of specific apoptosis measured in three independent experiments. *P* values were (1) <0.0001; (2) 0.0002; (3) non significant (ns); (4) ns; (5) 0.0003; (6) 0.0090. (D) Effect of P140 and 131–151 peptides on the viability of CD138^+^B220^−^ plasmocytes as measured by flow cytometry 20h after MRL/lpr PBLs treatment. Plots are representative of three independent experiments. (E) Viability of murine lymphoma T cells T29 studied by measuring mitochondrial reduction of 3-(4,5-dimethythiazol-2-yl)-5-(3-carboxymethoxyphenyl)-2-(4-sulfophenyl)-2H-tetrazolium dye 24h post-treatment with increasing concentrations of P140 peptide and two unrelated peptides X and Y used as positive controls. (F) Effect of P140 and 131–151 peptides on the B220 marker expression by CD4^+^ and CD8^+^ T cells. Expression of B220 was analyzed on these cells 20h after adding P140 or 131–151 peptides. The results are expressed as the mean percentage of cell subpopulations measured in three independent experiments. *P* values comparing CD4^+^B220^−^ and CD8^+^B220^−^ cells without or after P140 treatment were 0.059 and 0.0432, respectively. *P* values comparing CD4^+^B220^+^ and CD8^+^B220^+^ cells without or after P140 treatments were 0.0364 and 0.1707, respectively. The other comparisons were statistically non-significant.

We wondered whether the beneficial P140 effect was mediated by apoptosis of specific T cell subsets. We found *ex vivo* that after P140 co-incubation, in a peptide dose- and time-dependent manner, MRL/lpr PBLs displayed an apoptotic phenotype (annexin V positivity, reduction of DIOC_6_ dye uptake; Figure 3A and 3B; Figures S9, S10). Both MRL/lpr CD4^+^ and CD8^+^ T cells underwent specific apoptosis. Specific lysis of MRL/lpr CD4^+^ and CD8^+^ T cells was granzyme-B and caspase-dependent, while perforin inhibitor had very little effect (Figure 3C; inserts c1,c2). The number of viable B220^−^CD138/Syndecan-1^+^ plasma cells of the B cell lineage also decreased in the MRL/lpr PBL fraction (Figure 3D). Virtually no effect was observed when MRL/lpr PBLs were incubated with the non-phosphorylated or the ScP140 peptides, and no P140 effect was detectable with CBA/J PBLs (Figure 3A). No effect either was observed on murine CD4^+^CD8^+^ lymphoma T cells T29 (Figure 3E) and NIH 3T3 murine fibroblasts (not shown).

It is noticeable that after a 20h-incubation of PBLs with P140 peptide, the proportion of preapoptotic CD4^+^ activated blasts (bearing the B220/CD45R marker [19]) significantly increased in the culture (Figure 3F). No effect was measurable with peptide 131–151. The results shown with CD8^+^B220^+^ activated blasts were not statistically significant (Figure 3F).

### *In vivo*, P140 induces PBLs apoptosis via a mechanism mediated by γδ T cells

**Figure 4:**
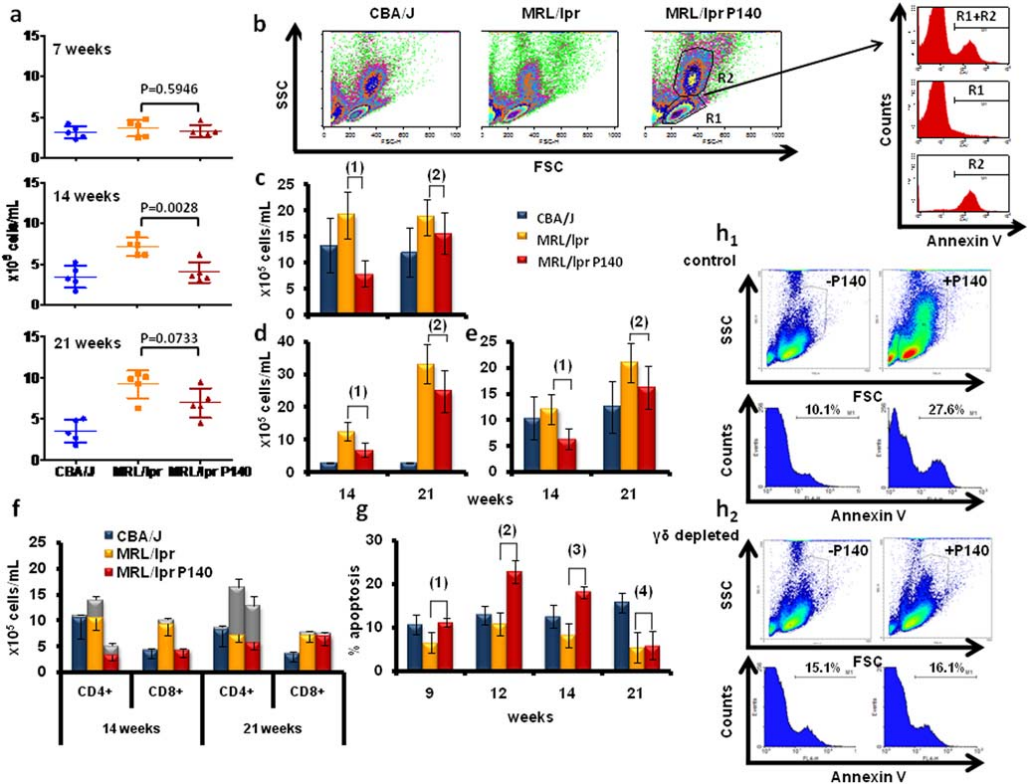
*In vivo* effect of P140 peptide on MRL/lpr PBL apoptosis. (A–H) Female MRL/lpr and CBA/J mice received peptide P140 intravenously (100 µg/mouse in saline) at weeks 5, 7, 9 and 13, according to our standard procedure [3]. Blue = CBA/J mice, orange = MRL/lpr mice, red = P140-treated MRL/lpr mice. (A) The number of leukocytes/ml was evaluated by counting cells at weeks 7, 14 and 21. Each symbol represents the absolute number of cells in one individual mouse (5 mice in each of the three groups). Horizontal bars represent the respective average values±SD. (B–F) Influence of P140 therapy on the cell distribution. Detailed data observed at weeks 9, 12, 14 and 21 weeks are shown in the Figure S11. (B) Different lymphocyte subpopulations present in the PBL fractions from CBA/J, MRL/lpr and P140-treated MRL/lpr mice evaluated by flow cytometry. The gates used in the studies are shown in the inserts. (C) Number of B220^−^TCRβ^+^ cells/ml in the peripheral PBL fraction collected from CBA/J, MRL/lpr mice and P140-treated MRL/lpr mice, respectively at 14 and 21 weeks of age. *P* values were (1) 0.0011; (2) ns. (D) Number of B220^+^TCRβ^+^ T cells/ml in the peripheral PBL fraction collected from CBA/J, MRL/lpr mice and P140-treated MRL/lpr mice, respectively at 14 and 21 weeks of age. *P* values were (1) 0.0084; (2) ns. (E) Absolute numbers of B220^+^TCRβ^−^ cells in the peripheral PBL fraction collected from CBA/J, MRL/lpr mice and P140-treated MRL/lpr mice, respectively at 14 and 21 weeks of age. *P* values were (1) 0.0059; (2) ns. (F) Number of CD4^+^ and CD8^+^ T cells/ml in the peripheral PBL fraction collected from CBA/J, MRL/lpr mice and P140-treated MRL/lpr mice, respectively at 14 and 21 weeks of age. The number of CD4^+^ and CD8^+^ T cells bearing the B220 marker is shown in gray. (G) Percentage of annexin V-allophycocyanin^+^ PBLs collected from 9, 12, 14 and 21 week-old CBA/J mice, MRL/lpr mice and P140-treated MRL/lpr mice. *P* values were (1) 0.0344; (2) 0.0051; (3) 0.0047; (4) ns, 0.8668. (H) PBL apoptosis measured after a single intravenous administration of P140 peptide into MRL/lpr mice that received control hamster IgG (H1) or depleting γδ T cell hamster IgG eight and three days before P140 treatment (H2). γδ T cell-depletion was achieved by two consecutive injections of 500 µg (1^st^ administration) and 1 mg (2^nd^ administration) of monoclonal antibody UC7-13D5 (half intraperitoneally and half intravenously). Sham depletion was performed with the same amount of hamster IgG isotype. Efficacy of γδ T cell depletion was measured by flow cytometry using anti-pan TCR γδ hamster monoclonal antibody GL3. Data reflect 3 mice per condition, and are representative of two separate experiments.

Next, we investigated *in vivo* the P140 effect on lymphocyte fate. It is well documented that compared to normal situation, MRL/lpr T and B cells are less sensitive to apoptosis, a feature resulting from defective activation-induced cell death, which is a FasL-dependent pathway [20]–[22]. It was therefore important to examine *in vivo* the effect of P140 administration on the apoptosis level of different lymphocyte subsets. After P140 administration into MRL/lpr mice according to our standard protection protocol [3], we found that at week 14 the absolute number of total peripheral white cells, the number of which is raised in MRL/lpr mice compared to CBA/J mice, was similar to the one measured in non-autoimmune mice (Figure 4A). At week 7 (i.e. at the onset of the disease) no difference was visible; at week 21 (eight weeks after the last administration) the effect was no longer statistically significant and this was true for all cell subsets axamined. The proportions of living (annexin V^−^) and apoptotic (annexin V^+^) cells were measured by FACS in haplotype-matched normal CBA/J mice, MRL/lpr prone mice and P140-treated MRL/lpr mice (Figure 4B). Compared to untreated MRL/lpr mice, the absolute number of TCRβ^+^B220^−^ and TCRβ^+^CD4^−^CD8^−^B220^+^ viable T cells, as well as that of B220^+^TCRβ^−^ viable B cell population, was diminished, at least until week 14 (Figure 4C–4E; Figure S11). Regarding peripheral viable CD4^+^B220^+^ T cell blasts and CD8^+^ T cell population, their absolute number, which are increased in 14-week-old MRL/lpr mice, clearly decreased in treated mice (Figure 4F).

In the PBL fraction the proportion of apoptotic cells (diminished in MRL/lpr mice, as expected) increased upon P140 peptide treatment (Figure 4G). This effect was no longer visible at week 21.

Previous studies have shown that HSC70 expressed on the cell surface of tumor cells might act as a loading molecule complexed with cellular peptides for presentation to certain immune effector cells, and notably to γδ T cells [9], [10]. Earlier studies also showed that γδ T cells interact with foreign and self-ligands, including MHC and MHC-like molecules and non-peptidic phosphoantigens [8], [23]. We thus tested a pathway in which P140 likely “presented” by HSC70 induces apoptosis of potentially harmful lymphocytes by a γδ T cell-mediated mechanism. Nine-week-old MRL/lpr mice received two successive intraperitoneal and intravenous administrations of anti-pan TCR γδ monoclonal antibodies UC7-13D5 [24] eight and three days before P140 treatment. Efficiency of depletion was analyzed by FACS using anti-pan TCR γδ antibody GL3 [25]. In these conditions, the number of viable γδ T cells dropped over 54% (3.5 to 1.6%) in the PBL fraction. The viable αβ T-cell compartment was marginally affected (62.7 to 69.2%). As shown in Figure 4H, while a single intravenous administration of P140 into MRL/lpr mice induced raised apoptosis of PBLs (27.6 vs. 10.1% annexin V^+^ cells; Figure 4H1), P140 induced no PBL apoptosis in γδ T cell-depleted MRL/lpr mice (16.1 vs. 15.1%; Figure 4H2). Similar results were obtained in two independent experiments including each three mice per group. These data clearly indicate that *in vivo* P140 induces PBL apoptosis *via* a mechanism involving γδ T cells.

### Several genes involved in inflammation are rapidly down-regulated in CD4^+^ T cells from P140-treated mice

**Figure 5:**
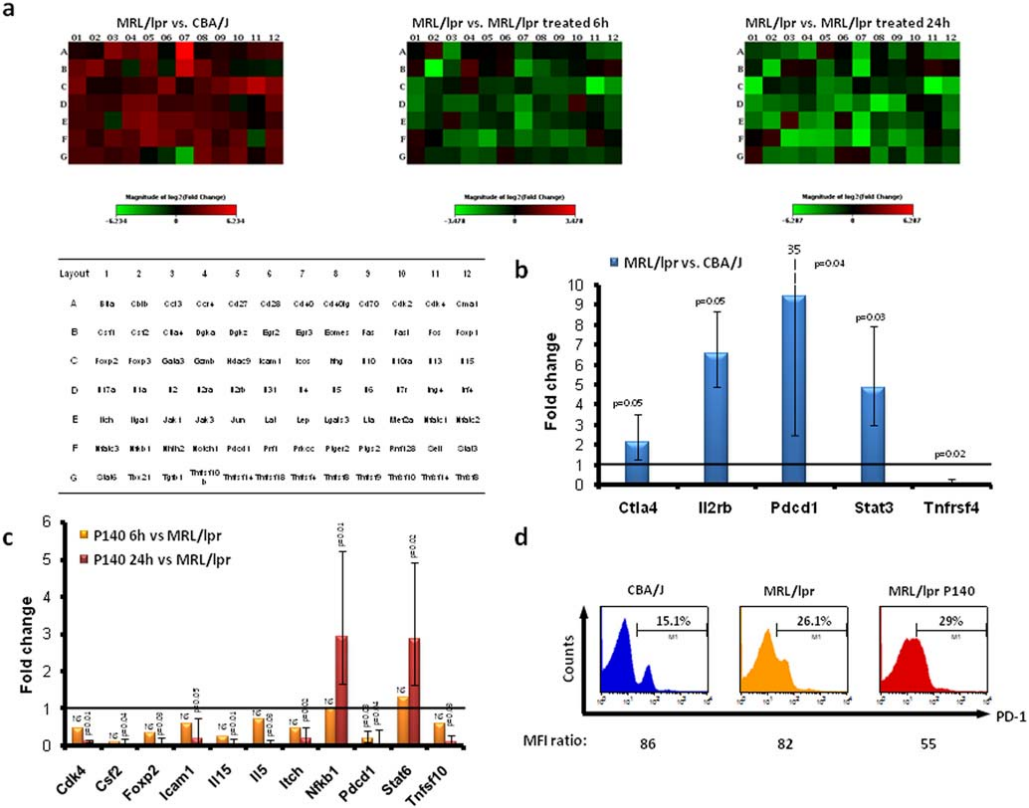
P140-induced changes in expression of inflammation/tolerance/anergy-related genes in MRL/lpr mice. T cell anergy and tolerance RT-PCR-Array were used to evaluate expression of a selected set of genes expressed by non-activated T CD4^+^ cells from 6 week-old CBA/J mice, MRL/lpr mice and P140-treated MRL/lpr mice. (A) Heat map of differentially-expressed genes between 6-week-old normal CBA/J and MRL/lpr mice and between non-treated MRL/lpr and MRL/lpr mice that received each a single intravenous injection of 100 µg P140 peptide (evaluated 6 h and 24 h post-administration). A scale of colour intensity is presented for each heat diagram. Red, up-regulation; green, down-regulation; black, no change. Genes included in the microarray are listed in the table. Results are the average in gene expression for four independent experiments, with three mice per group in each experiment. (B) Changes in gene expression between non-activated T CD4^+^ cells from CBA/J and non-treated MRL/lpr. Out of the 84 genes tested, four were significantly up-regulated and one significantly down-regulated. The other changes were not statistically-significant. (C) Changes in gene expression between non-activated T CD4^+^ cells purified from non-treated MRL/lpr mice and MRL/lpr mice collected 6 and 24h post-treatment. (D) PBL surface expression of PD-1 studied in the three groups of mice by flow cytometry. Mean fluorescence intensity (MFI) values are indicated.

In previous studies we found that P140 administration into MRL/lpr mice transiently abolished T cell responsiveness to self-CD4^+^ T cell epitopes present in various spliceosomal proteins [5], [6]. Our experiments were thus designed to test whether *in vivo* exposure to P140 might regulate expression of genes associated with T cell tolerance and anergy. qPCR and dedicated arrays were used to profile the expression of 84 genes related to T cell tolerance (Figure 5A). Total RNA was extracted from LN non-activated (B220^−^) CD4^+^ T cells (6-week-old mice) purified by negative selection. Five house-keeping genes were used to normalize the results and the whole experiment was repeated four times. Compared to CBA/J CD4^+^ T cells, pdcd1, il2rb, stat3 and ctla4 gene expression was significantly up-regulated and tnfrsf4 gene expression significantly down-regulated in MRL/lpr CD4 T^+^ cells of the same age (Figure 5A and 5B). Expression of the 79 other genes was not significantly affected (*P*>0.05). After a single intravenous administration of P140 into MRL/lpr mice, expression of several genes was down-regulated (Figure 5A and 5C). Thus, pdcd1 gene expression, which was increased in MRL/lpr mice, was significantly down-regulated 6h only after P140 administration (*P* = 0.03). This feature was confirmed by FACS in showing an increased number of CD4^+^ T cells expressing PDCD1/PD-1 in MRL/lpr mice, as compared to CBA/J mice (Figure 5D), and a decreased expression level of PDCD1/PD-1 subsequent to a single P140 injection into MRL/lpr mice (Figure 5D). Post-P140 injection, other genes were either significantly down-regulated (il5 and il15, csf2, tnfsf10, cdk4, icam1, foxp2, and itch) or up-regulated (NFκb1 and stat6) in MRL/lpr mice (Figure 5C).

## Discussion

**Figure 6:**
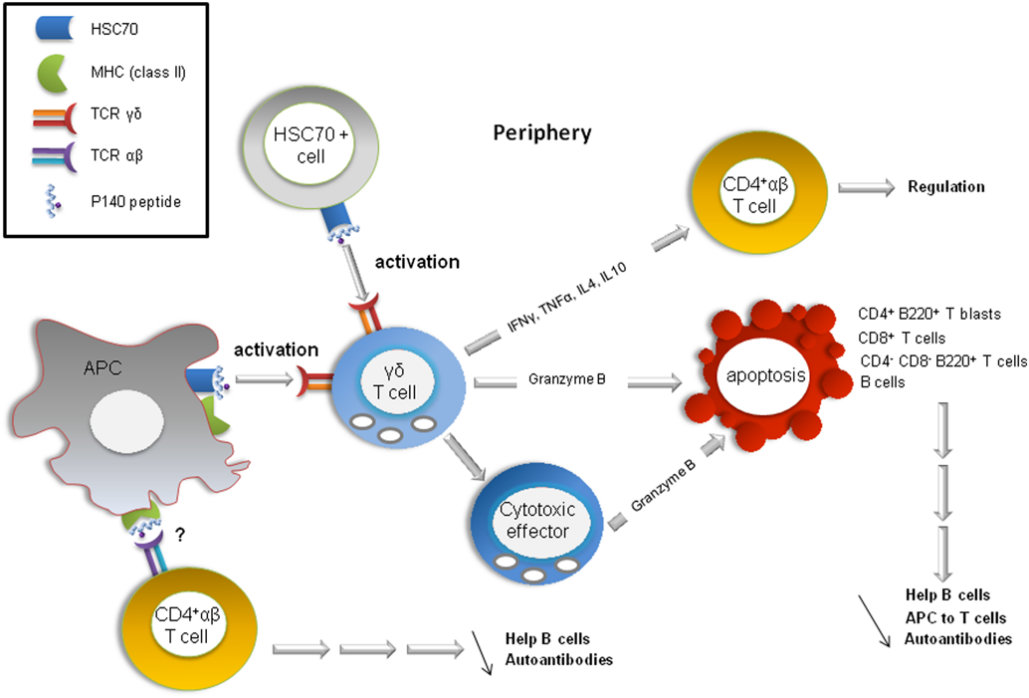
Proposed mechanisms underlying P140 peptide protective effect against disease in MRL/lpr mice. P140 physically associates with a unique receptor, the constitutive HSC70 chaperone protein, which is present on a majority (if not all) of immune cells. The P140-HSC70 complex (but not the putative complex encompassing the non-phosphorylated peptide 131–151) directly or indirectly activates γδ T cells. *Via* a granzyme B- and caspase-dependent mechanism, apoptosis targeting activated CD4^+^ T blasts, CD8^+^ T cells, CD4^−^CD8^−^B220^+^ T cells and B cells is induced (directly or via another cytotoxic effector) leading to a diminution of T-cell help to B cells, a diminution of antigen-presenting cell activity by B cells and a drop of antibody production by autoreactive B cells. In parallel, activated γδ T cells secrete cytokines that modulate the αβ T cell fate. This model does not exclude the possibility that in parallel, P140 also induces anergy, deletion, or deviation of autoimmune T cells, *via* antagonism or partial agonism of the αβ Τ lymphocytes TCR [3]. In addition, P140 acts on non-activated CD4^+^ T cells by altering several intracellular pathways, leading, in particular, to dramatic effects on the NF*k*B and TNF pathways, and diminution of PD-1 expression, which is raised in MRL/lpr mice.

This report defines a previously undescribed pathway taken by a peptide to immunomodulate autoimmune response (diagrammed in Figure 6). Key features of this pathway, as identified in MRL/lpr mice, are (i) P140 binding to a unique receptor, the constitutively-expressed HSC70 chaperone protein; (ii) involvement of γδ T cells in a granzyme B- and caspase-dependent mechanism, leading directly or indirectly via cytotoxic effectors to apoptosis of distinct (activated) T and B lymphocytes/blasts. In parallel, an important effect is also observed on non-activated CD4^+^ T cells, as shown at the level of several genes involved in inflammation, which are rapidly down- or up-regulated upon P140 treatment. Several check-points are decisive in this scheme and might explain the remarkable protecting efficacy of P140 in lupus. First, HSC70 but not inducible Hsp70 seems to operate as a primary receptor. Interestingly, the immunosuppressive compound 15-deoxyspergualin has been shown to specifically bind to HSC70, which among other effects inhibits NF-κB nuclear translocation. It suppressed the progression of polyclonal B cell activation and lupus nephropathy in MRL/lpr mice [26]. *Ex vivo*, it altered the effective presentation of antigen to T cells by antigen-presenting cells and changed the levels of cytokines produced after engagement [27]. In a short trial, two of three treated SLE patients showed non-severe infectious episodes after 15-deoxyspergualin treatment [28].

Both P140 and 131–151 peptides (but not ScP140) bind to HSC70 showing that the phosphoryl moiety does not alter recognition and that sequence specificity exists. It has been shown that HSC70 undergoes conformational changes upon binding peptides [29]. An interesting issue would be to determine if HSC70 displays different conformations upon binding P140 and 131–151 peptides with a possible effect on recognition properties of the resulting complex. HSC70 plays an important role in binding and protecting peptide for MHC class II molecules [30]. It interacts with MHC molecules and particularly with HLA-DR4 [31]. Whether this association is of central importance remains to be determined.

If the phosphoryl moiety has no obvious effect on the recognition of peptide 131–151 by HSC70, it apparently represents an important check-point in the P140 pathway for γδ T cell recognition. Thus, only P140 phosphoantigen presented in the context of HSC70 could activate γδ T cells. It is not known yet if all γδ T cell subsets can be activated by this new ligand. γδ T cells present an altered homing in lupus patients with an increased level in clinically normal skin and a decreased level at the periphery, which in some studies correlate with disease activity [32]–[34]. MRL/lpr mice lacking γδ T cells develop more severe disease [11]. Restoring γδ T cell functions might therefore constitute an important step [35]. Upon administration of P140 into MRL/lpr mice, activated T and B cells as well as DN T cells were found to be engaged in an apoptotic process. This observation is crucial in the P140 pathway since early mortality of MRL/lpr mice is associated to T cell lymphoaccumulation reminiscent of *lpr*-related lymphoproliferative disease. How γδ cell-mediated P140-induced killing precisely occurs remains to be elucidated. *Ex vivo,* this Fas/FasL-independent process involves granzyme-B and caspases but apparently not perforin. Knowing that γδ T cells express CD40, an important co-stimulation might also occur *via* CD40L(CD154), which is expressed in a sustained manner at the surface of lupus CD4^+^ T cells and B cells [36]. CD16(FcγRIII), the low affinity Fc receptor for IgG expressed by γδ T cells (and not by αβ T cells), may also play an important role; it was shown, for example, that soluble CD16 administered to lupus mice after the onset of disease has a beneficial effect on their biological and clinical behavior [37].

Although the absolute number of γδ T cells is low in normal and lupus conditions, this small population of regulatory cells exerts numerous central effects [8], [23]. Thus, upon activation, they might influence dendritic cells, NK cells, αβ T cells, and *via* either contact-dependent mechanisms or chemiokine/cytokine secretion, might contribute to tolerance restoration in P140-treated mice. Beside this mechanism, which affects activated lymphocytes, P140 also alters properties of non-activated CD4^+^ T cells. Thus, hyperexpression in MRL/lpr mice of PD-1(CD279) molecule, a member of the CD28 family, which is a hallmark of activated T cells [38], is reversed in P140-treated mice. After P140 treatment other genes were significantly up-regulated, such as genes coding for STAT6 that promotes Th2-cell differentiation and NFκB1/p50, or down-regulated, such as genes coding for adhesion molecule ICAM-1, IL-15, IL-5 and CFS2 cytokines, transcriptional regulator FOXP2, CDK4 (blocking the formation of cyclin-D/CDK4 complexes in murine lupus was shown to inhibit antigen-induced and anti-CD3-mediated splenic T cell proliferation as well as T-dependent B cell activation and proliferation in vitro [39]), the E3 ligase Itch, and TNFSF10(TRAIL) (a member of the TNF ligand superfamily, which exacerbates lupus [40]). Although additional work must be done at the molecular level to understand the precise signaling pathways induced upon P140 administration, these results show that *in vivo* P140 exerts a rapid effect on genes involved in inflammation and can restore some dysfunctions, as exemplified with PD-1.

In conclusion, we have demonstrated that in MRL/lpr mice P140 binds to HSC70 and induces a novel regulatory circuit involving γδ T cells, which affects autoreactive T and B cell survival. We thus propose a comprehensive mechanism triggered by P140 to control the lupus disease in this mouse strain. In addition, these findings shape our understanding of the immunoregulatory functions of γδ T cells in autoimmune diseases.

## Materials and Methods

### Synthetic peptides

The synthesis and purification of P140 peptide (RIHMVYSKRpSGKPRGYAFIEY; produced under Good Manufacturing Practice conditions), and of peptides 131–151 (RIHMVYSKRSGKPRGYAFIEY) and ScP140 (YVSRYFGpSAIRHEPKMKIYRG) were described previously [1], [3], [7]. For the biotin-labeled peptides, the biotin moiety was introduced at the end of peptide assembly as a Fmoc-Lys(biotin)-OH derivative. Homogeneity of peptides was checked by analytical high-performance liquid chromatography, and their identity was assessed by matrix-assisted laser desorption and ionization time-of-flight (TOF) mass spectrometry using a Protein TOF apparatus (Bruker Spectrospin). The solubility limits of both peptides in the cell culture medium RPMI 1640, PBS and distilled water were determined at 20 and 37°C using dynamic light scattering measurements [18].

### ^1^H-NMR experiments

D_2_O (99.8%) and trimethylsilyl-3-propionic acid-2,2,3,3-d_4_ (TSP-d_4_) were purchased from Euriso-top. Peptides 131–151 and P140 (2 mM final) were dissolved in 0.6 mL of 95% H_2_O and 5% D_2_O. The pH of the NMR sample was adjusted to 4.0. Spectra were recorded at 4°C using a Bruker DRX600 NMR spectrometer. All chemical shifts were referenced to methyl signals of TSP-d_4_ as an internal standard. 2D TOCSY experiments [41], [42] were performed on each peptide with a mixing time, τ_m_, of 20 and 70 ms. Four 2D-NOESY experiments with mixing times ranging from 100 to 500 ms were performed. The build-up curve [43] for different NOE correlations showed that spin diffusion was negligible for τ_m_ = 200 ms. The spectral width in F1 was 7200 Hz. Water resonance suppression in the TOCSY and NOESY experiments was achieved using the water suppression by gradient-tailored excitation (WATERGATE) sequence [44], [45]. Data processing was performed using XWIN-NMR software. Distance restraints for structural calculations were generated from the NOESY spectra (τ_m_ = 200 ms) using XEASY software [46] and by classifying NOE cross-peaks as strong (1.8–2.5 Å), medium (2.5–3.5 Å) and weak (3.5–5.5 Å) based on the volume of the respective cross-peaks [47], calculated by taking as reference the distance of 1.78 Å between two non-equivalent geminal protons.

### Molecular dynamics calculation

Energy minimization (EM) and molecular dynamics (MD) calculations were performed on an Octane Silicon Graphics workstation. The structure calculations were done by restrained molecular dynamics in dihedral angle space using the standard simulated annealing protocol, as implemented in the program DYANA-1.4 [48] to generate, in a random way, a set of 100 initial structures corresponding to the NMR constraints. The SA protocol permits the exploration of the whole conformational space to find the global minimum energy conformation of the molecule and it has been shown to be a useful procedure for the study of constrained systems [49]–[51]. Fifty structures with the lowest energies were chosen for the refinement step with restrained minimization. Each of them was submitted to 900 steps of minimization (300 steps with steepest descent, 300 steps with conjugate gradient and 300 steps with Newton-Raphson methods), then to 35ps of MD *in vacuo* at 300K for equilibration and finally to 200ps of MD applying derived NMR restraints with a force constant of 20 kcal mol^−1^ Å^−2^. Conjugate gradient EM was completed within 750 steps. The 50 refined structures were evaluated with MOLMOL [52] as results. For MD and EM calculations, the DISCOVER-3 module of the Insight II program (Accelrys, Inc.) was used. Consistent-valence force field with a default cut-off distance of 9.5 Å and a distance-dependent dielectric constant, equal to 4r, was applied to all peptides [53]. In order to avoid structural artefacts due to overestimation of coulombic interactions *in vacuo*, the net electric charges of the N- and C-terminal charged groups were neglected. The φ angle for the non-glycine L-residues was constrained between −175° and 0°.

### Identification of P140 receptor

The detection of cell surface protein(s) recognized by P140 was adapted from a previously described method [54], [55]. Total splenocytes and LN cells from 7–8 week-old MRL/lpr or unprimed CBA/J mice were isolated on nylon membrane (100 µM). Cells (about 50×10^6^) were incubated for 1h at room temperature (RT) in 5 mL of serum free RPMI 1640 culture medium (Biomedia) containing biotin-labeled peptides (P140 peptide, peptide 131–151 or scramble P140 peptide). In some experiments, unlabeled P140 peptide was added as a competitor. After washing cells once in phosphate buffered-saline (PBS) containing 1 mM EDTA, nucleus-free cell extracts were prepared in lysis buffer E (20 mM Tris-HCl pH 7.5, 50 mM NaCl, 5 mM MgCl_2_, 1 µL/1mL protease inhibitors from Sigma, 0.5% Triton X100, 1 mM NaF, 1 mM Na_3_VO_4_). The complexes formed between the biotin-labeled P140 and putative cell-surface receptors were isolated by purification of the extracts using 100 µL avidin-agarose (ImmunoPure Immobilized Avidin, Pierce) in PBS containing 1 mM EDTA, 1mM NaF, and 1 mM Na_3_VO_4_. After overnight incubation at 4°C, the samples were washed extensively with the same buffer. The purified proteins were subjected to SDS-PAGE and revealed by colloidal blue staining. The relative signal intensity of each band was quantified by densitometry with the ImageJ 1.33u freeware (Gel Analyzer tool, NIH) after scanning. The identification of HSC70 was deduced from mass spectrometry analysis as described below after cutting bands of interest.

### Protein preparation for in-gel digestion

The gel pieces were successively washed with 50 µL of 25 mM NH_4_HCO_3_ and 50 µL of acetonitrile (three times), and dehydrated with 100 µL acetonitrile before reduction in the presence of 10 mM dithiothreitol in 25 mM NH_4_HCO_3_ (1h at 57°C) and alkylation in the presence of 55 mM iodoacetamide in 25 mM NH_4_HCO_3_. For tryptic digestion, the gel pieces were resuspended in 2 volumes of trypsin (12.5 ng/µL; Promega V5111) freshly diluted in 25 mM NH_4_HCO_3_ and incubated overnight at 37°C. The digested peptides were then extracted from the gel in a solution containing 34.9% H_2_O, 65% acetonitrile and 0.1% HCOOH, and directly analyzed by nanoLC-MS/MS.

### Chromatography conditions on NanoAcquity

The analysis was performed using a nanoACQUITY Ultra-Performance-LC (UPLC; Waters). The samples were trapped on a 20×0.18 mm, 5 µm Symmetry C18 precolumn (Waters), and the peptides were separated on a ACQUITY UPLC® BEH130 C18 column (Waters), 75 µm×200 mm, 1.7 µm particle size. The solvent system consisted of 0.1% formic acid in water (solvent A) and 0.1% formic acid in acetonitrile (solvent B). Trapping was performed during 3 min at 5 µL/min with 99% of solvent A and 1% of solvent B. Elution was performed at a flow rate of 400 nL/min, using 1–40% gradient (solvent B) over 35 min at 45°C followed by 65% (solvent B) over 5 min.

### MS and MS/MS conditions on SYNAPT mass spectrometer

The MS and MS/MS analyzes were performed using a SYNAPT™ apparatus, an hybrid quadrupole orthogonal acceleration time-of-flight (TOF) tandem mass spectrometer (Waters) equipped with a Z-spray ion source and a lock mass system. The capillary voltage was set at 3.5 KV and the cone voltage at 35 V. Mass calibration of the TOF was achieved using phosphoric acid (H_3_PO_4_) on the [50;2000] m/z range. Online correction of this calibration was performed with Glu-fibrino-peptide B as the lock-mass. The ion (M+2H)^2+^ at m/z 785.8426 was used to calibrate MS data and the fragment ion (M+H)^+^ at m/z 684.3469 was used to calibrate MS/MS data during the analysis.

For tandem MS experiments, the system was operated with automatic switching between MS and MS/MS modes (MS 0.5 s/scan on m/z range [250;1500] and MS/MS 0.7 s/scan on m/z range [50;2000]). The three most abundant peptides (intensity threshold 60 counts/s), preferably doubly and triply charged ions, were selected on each MS spectrum for further isolation and CID fragmentation with 2 energies set using collision energy profile. Fragmentation was performed using argon as the collision gas. The complete system was fully controlled by MassLynx 4.1 (SCN 566; Waters).

### Data analysis and protein identification

Raw data collected during nanoLC-MS/MS analyses were processed and converted with ProteinLynx Browser 2.3 (Waters) into .pkl peak list format. Normal background subtraction type was used for both MS and MS/MS with 5% threshold and polynomial correction of order 5, and deisotoping was performed. The MS/MS data were analyzed using the MASCOT 2. 2. 0. algorithm (Matrix Science) to search against the UniProtKB/Swiss-Prot database, version 54.8, concatenated with reversed copies of all sequences (2×349,480 entries). Spectra were searched with a mass tolerance of 15 ppm for MS and 0.07 Da for MS/MS data, allowing a maximum of one missed cleavage site by trypsin and with carbamidomethylation of cysteine residues and oxidation of methionine residues specified as fixed and variable modifications, respectively. Protein identifications were validated when at least two peptides were found with Mascot ion score greater than 35 for each MS/MS spectrum.

For the estimation of the false positive rate in protein identification, a target-decoy database search was performed. Criteria used for protein identifications followed the general guidelines for reporting proteomic experiments (MIAPE; http://www.psidev.info).

### Surface plasmon resonance analysis

BIAcore 3000 system (Biacore AB) was used to evaluate the binding of P140 peptide to HSC70 protein. Sensor chip CM5, surfactant P20, amine coupling kit containing N-hydroxysuccinimide (NHS) and N-ethyl-N′-dimethylaminopropyl carbodiimide (EDC), 2-(2-pyridinyldithio)ethaneamine (PDEA) and ethanolamine were from Biacore AB. Biosensor assays were performed with HBS-EP buffer as running buffer (10 mM HEPES, 150 mM NaCl, 3 mM EDTA, 0.005% surfactant P20, pH 7.4). The different compounds were diluted in the running buffer. The sensor chip surface was regenerated after each experiment by injecting 10 µL of 10 mM HCl. Recombinant bovine HSC70 (Stressgen) was immobilized on flow cells of a CM5 sensor chip through its thiol groups using 35 µL PDEA in 50 mM borate buffer, pH 8.3 on the NHS/EDC-activated matrix. Then, 35 µL of HSC70 (100 µg/mL in formate buffer, pH 4.3) were injected until a response of 13,000 response units (RU) corresponding to 13 ng/mm^2^ of HSC70 was immobilized. Twenty µL of a 50 mM cysteine/1 M NaCl solution was used to saturate unoccupied sites on the chip. The direct binding measurement of P140 peptides to HSC70 was carried out at 25°C with a constant flow rate of 20 µL/min. P140 peptide and analogues were injected in the flux at different concentrations for 3 min, followed by a dissociation phase of 3 min. The kinetic parameters were calculated using the BIAeval 3.1 software on a personal computer. Analysis was performed using the simple 1∶1 Langmuir binding model. The specific binding profiles were obtained after subtracting the response signal from the control empty channel and from blank-buffer injection. The fitting to each model was judged by the χ square value and randomness of residue distribution compared to the theoretical model.

### Immunofluorescence

To study the co-localization of HSC70 and P140 peptide staining, freshly isolated splenocytes from MRL/lpr mice were washed with PBS and incubated at RT for 45 min in PBS containing 25 µM biotinylated P140. Cells were then washed three times in Tris buffered-saline (TBS) and fixed for 1 h at RT in 4% paraformaldehyde (PFA) in 0.1M phosphate buffer (PB). Fixed cells were incubated for 1h at RT with a rat monoclonal anti-HSC70 antibody labeled with R-phycoerythrin (PE; 1∶150; clone 1B5; Stressgen) in TBS containing 2% (w/v) BSA. After washing in TBS, cells were incubated with fluorescein isothiocyanate (FITC)-labeled streptavidin (1∶400, Molecular Probes) and 4′,6′-diamidino-2-phenylindole (DAPI, 1∶1,000; Molecular Probes), washed thrice in TBS and fixed for additional 1 h at RT in 4% PFA in 0.1M PB. Control experiments were performed by omitting biotinylated peptide. Samples were finally mounted on slides using Dako's fluorescence mounting medium. Pictures were obtained with a Zeiss fluorescence microscope (Axiovert 200 M) using an X63 magnification. Images were acquired with a digital camera using Axiovision 4.37 software (Zeiss). Double immunofluorescence images were obtained by superimposing two single-color pictures with Adobe Photoshop 8.0. To study the localization of HSC70 in permeabilized cells, see the legend of the Figure S2.

### Immuno-electron microscopy

To study the subcellular localization of HSC70 by immunoelectron microscopy, freshly isolated PBLs from MRL/lpr mice were washed with PBS and fixed in 4% (v/v) PFA and 0.2% (v/v) glutaraldehyde in 0.1M PB for 1h at RT. Cells were embedded in 1% agarose, permeabilized with 0.2% saponin for 10 min, washed and saturated with 2% BSA and 0.2% (w/v) water fish skin gelatin (WFSG, Sigma) in TBS before overnight incubation at 4°C with anti-HSC70 rat monoclonal antibody (1∶200; clone 1B5; Abcam) in TBS containing 0.2% BSA and 0.05% WFSG. Bound antibody was visualized by preembedding labeling using goat anti-rat IgG conjugated to 0.8 nm ultra-small gold particles (Aurion) in TBS containing 2% acetylated BSA (Aurion) and 0.2% WFSG. After washing in TBS, cells were fixed in 1% glutaraldehyde, washed again in TBS, and gold particles were enhanced using a silver kit (HQ silver; Nanoprobes). After further washing in 0.1M PB, cells were postfixed for 10min in 0.5% OsO4, dehydrated in graded series of ethanol, treated with propylene oxide and embedded in epon (Inland Europe). Ultrathin sections were stained with uranyl acetate and lead citrate and examined by TEM using a Hitachi H600 microscope. Images were acquired using a CCD camera (Hamamatsu).

### Cellular assays and FACS analysis

Lymphocytes from normal and lupus mice were purified by density separation (Lympholyte-M, d = 1.0875; Cedarlane). Peripheral blood lymphocytes (PBLs) were collected, washed 3 times in L-alanyl-L-glutamine-enriched RPMI 1640 medium (Biomedia) containing 10% fetal calf serum (FCS; Biomedia), 10 µg/mL gentamycin, 10 mM HEPES, and 5×10^−5^ M β-mercaptoethanol, and resuspended at 1×10^6^ cells/mL in the above-described medium. Cell death occurring in the presence of peptides was measured in triplicate in a time scale of 30 min to 20 h using 1×10^5^ cells and 0–80 µM peptide/well. Apoptosis was measured by double staining with allophycocyanin (APC)-labeled annexin V (BD Bioscience) and propidium iodide using a FACSCalibur flow cytometer (BD Biosciences) [56]. Apoptosis was also studied by a decrease in mitochondrial transmembrane potential as detected by the reduction of DiOC6(3) dye uptake [56]. For specific labeling the following antibodies were purchased from BD Bioscience: FITC-conjugated-B220- and TCRβ-antibodies; PE-conjugated-CD4- and CD138-antibodies; PerCP-conjugated-CD8- and B220-antibodies. FITC-conjugated TCRγδ and PD-1 antibodies were purchased from eBioscience. PE-conjugated-HSC70-antibodies were purchased from Stressgen. For testing the influence of apoptosis inhibitors, the following inhibitors were incubated with cells for 30 min at 37°C: Granzyme B inhibitor I (20 µMZ-AAD-CMK; Calbiochem), perforin inhibitor (5 nM concanamycin A; Calbiochem) and caspases inhibitors (20 µM Z-VAD-FMK; Promega). Data acquisition and analysis were performed using BD Bioscience CellQuest 3.3 software. Depending on the cell subsets studied, between 20,000 and 100,000 events were acquired. Experiments were repeated at least 3 times. Data are presented as mean±SD. Statistical significance was determined by Student's *t*-test. Differences were considered significant at *P* <0.05.

### FACS analysis of cell surface HSC70 expression

Splenocytes were washed in PBS and incubated for 20 min at 4°C with biotinylated peptide diluted in PBS containing 2% (v/v) FCS at the indicated concentrations. After washing, cells were incubated with APC-labeled streptavidin at 1 µg/mL in PBS containing 2% (v/v) FCS at 4°C for 15 min before another wash and subsequent analysis by flow cytometry. At least 20,000 events were acquired.

### Transcriptomic assays

For monitoring the effect of P140 peptide on the expression of focused gene panels linked to inflammation, P140 peptide (or PBS as control) was administered intravenously into MRL/lpr and CBA/J mice (100 µg in PBS/mouse/injection), and 6 and 24 h post-treatment, T CD4^+^ cells mRNA levels for 84 genes involved in T cell anergy and 5 constitutive genes were measured using the SuperArray quantitative PCR assay (SuperArray Cell Anergy and Tolerance RT2 Profiler™ PCR Array, Bioscience Corp). Briefly, inguinal, popliteal, and periaortic LN from 6-week-old MRL/lpr or CBA/J mice were removed 6 or 24 h after intravenous injection with 100 µg P140 in PBS (3 mice) or PBS alone and washed in L-alanyl-L-glutamine enriched RPMI 1640-Glutamax I (Life Technologies) containing 10% FCS, 10 mg/mL gentamicin, 10 mM HEPES, and 5×10^−5^ M 2-mercaptoethanol. LN cells were then enriched for CD4^+^ T cells by negative selection. LN cells were depleted of macrophages, granulocytes, B cells, and CD8^+^ T cells by incubation with anti-CD11b (Mac-1), anti-GR1 (8C5), anti-CD19 (1D3), anti-B220 (6B2) and anti-CD8 (Lyt-2) monoclonal antibody purified in-house and with magnetic beads coupled to anti-rat immunoglobulins (Dynal). After negative selection, greater than 95% of the resulting cells were CD4^+^ positive by fluorescence-activated cell sorting analysis.

Total RNA was extracted from approximately 5×10^6^ T CD4^+^ cells using Qiagen RNeasy® mini kits with inclusion of a DNase treatment step. First strand cDNA synthesis and quantitative real-time PCR with SYBR green master mix was performed in Stratagene MX4000 Real Time PCR System®. In brief, cDNA was prepared from 500 ng total RNA by using a RT2 PCR array first strand kit. A total volume of 25 µL of PCR mixture, which included 12.5 µL of RT2 Real-Time SYBR Green/ROX PCR master mix from SuperArray Bioscience containing HotStart DNA polymerase, SYBR Green dye, and the ROX reference dye (Tebu-bio), 11.5 µL of double-distilled H_2_O, and 1 µL of template cDNA, was loaded in each well of T cells Anergy and Tolerance RT2 Profiler PCR array (SuperArray Bioscience®), according to the manufacturer's instructions. PCR amplification was conducted with an initial 10 min step at 95°C followed by 45 cycles of 95°C for 15 s and 60°C for 1 min. The fluorescent signal from SYBR Green was detected immediately after the extension step of each cycle, and the cycle at which the product was first detectable was recorded as the cycle threshold. Data were imported into an Excel database and analyzed using the comparative cycle threshold method with normalization of the raw data to housekeeping genes including β-glucuronidase, hypoxanthine guanine phosphoribosyl-transferase1, β-actin, Hsp90 and glyceraldehyde-3-phosphate dehydrogenase. Data were expressed as the mean±SD of four independent experiments.

### *In vivo* depletion of γδ T cells

Hamster anti-pan TRC γδ monoclonal antibodies (UC7-13D5 clone) were purified from hybridoma culture supernatants using HiTrap protein G column (Pharmingen). γδ T cell-depletion was achieved by two consecutive injections of hamster monoclonal antibodies (500 µg for the first administration and 1mg for the subsequent one; half intraperitoneally and half intravenously) 8 and 3 days before P140 administration. Sham depletion was performed with the same amount of hamster IgG isotype (The Jackson Laboratory). MRL/lpr mice received P140 peptide (100 µg/mouse) in saline *via* the intravenous route [3]. Flow cytometry was used to monitor γδ T cell depletion and αβ T cell number, and to measure PBL apoptosis.

### Animal welfare

All experimental protocols were carried out with the approval of the local Institutional Animal Care and Use Committee (CREMEAS).

## Supporting Information

Figure S1 (0.18 MB TIF)

Figure S2 (0.14 MB TIF)

Figure S3 (0.31 MB TIF)

Figure S4 (0.12 MB TIF)

Figure S5 (0.24 MB TIF)

Figure S6 (0.24 MB TIF)

Figure S7 (0.16 MB TIF)

Figure S8 (0.07 MB TIF)

Figure S9 (0.08 MB TIF)

Figure S10 (0.19 MB TIF)

Figure S11 (0.43 MB TIF)

Text S1 Supporting Results
(0.07 MB DOC)

Table S1 (0.03 MB DOC)

Table S2 (0.03 MB DOC)

Table S3 (0.04 MB DOC)

Table S4 (0.03 MB DOC)
