# Supplementary figures and images for: The Spliceosomal Phosphopeptide P140 Controls the Lupus Disease by Interacting with the HSC70 Protein and via a Mechanism Mediated by γδ T Cells
Source: PLoS One. 2009 Apr 23;4(4):e5273. doi: 10.1371/journal.pone.0005273 (PMC2669294; doi:10.1371/journal.pone.0005273)

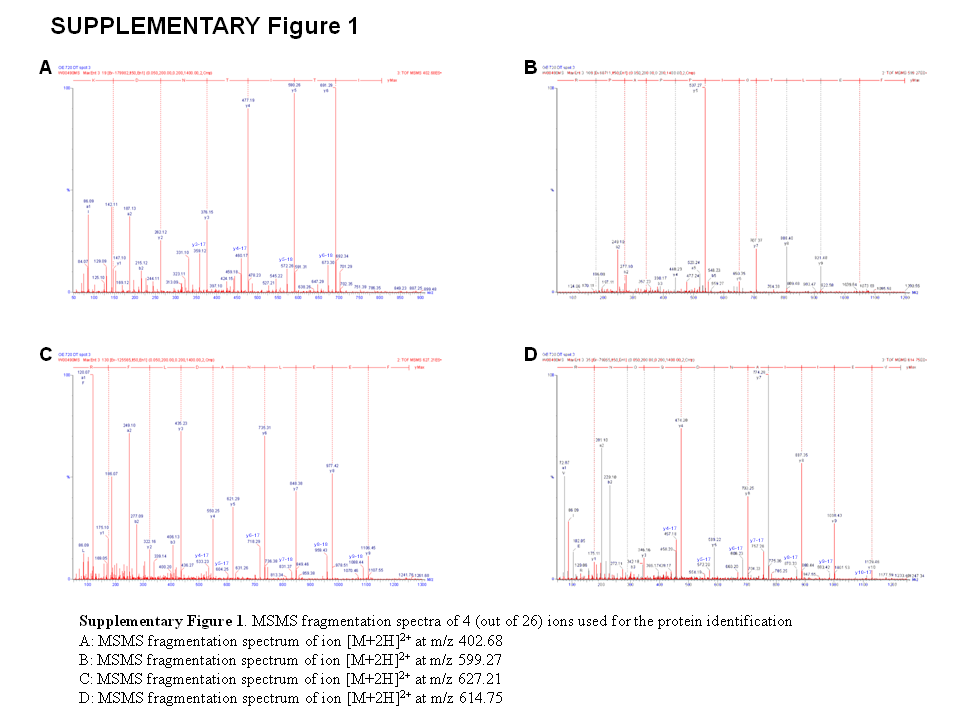

Supplement: Figure S1 — (0.18 MB TIF) [file pone.0005273.s001.tif]

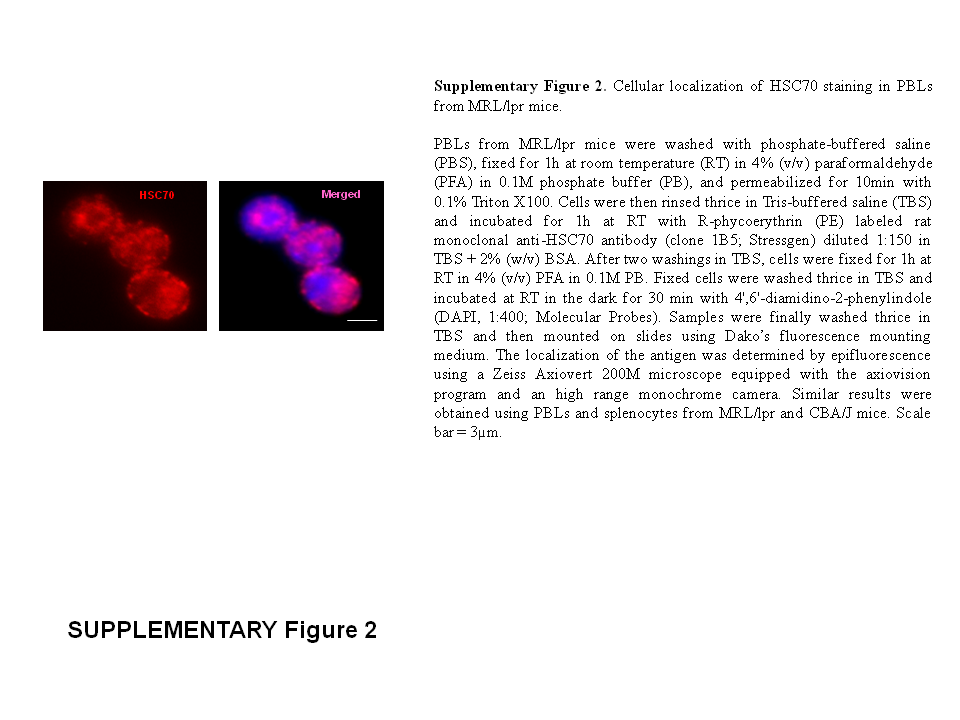

Supplement: Figure S2 — (0.14 MB TIF) [file pone.0005273.s002.tif]

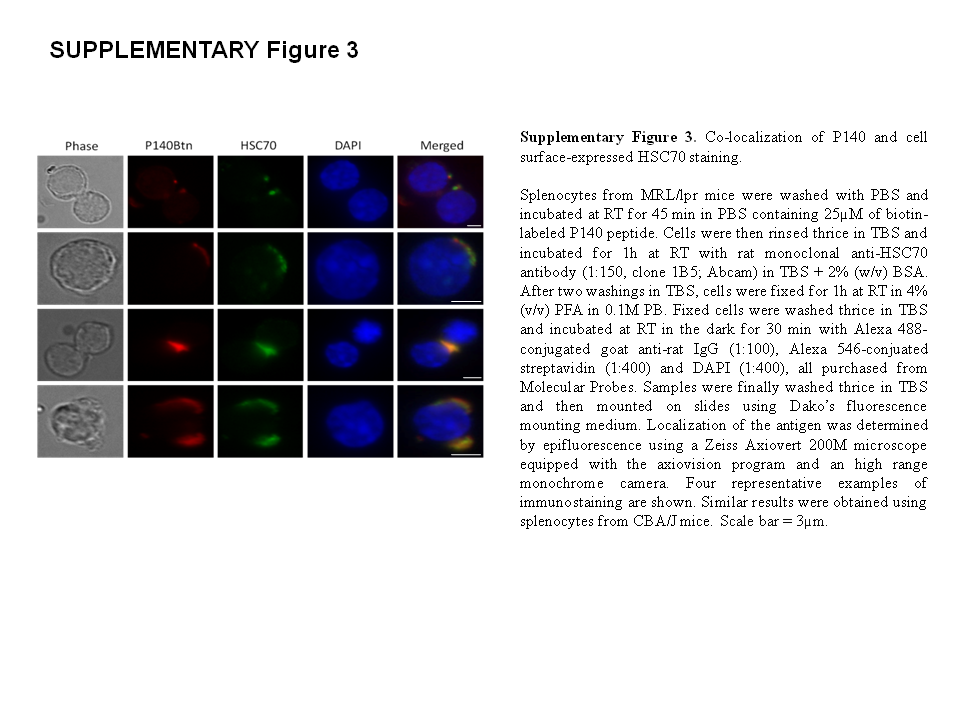

Supplement: Figure S3 — (0.31 MB TIF) [file pone.0005273.s003.tif]

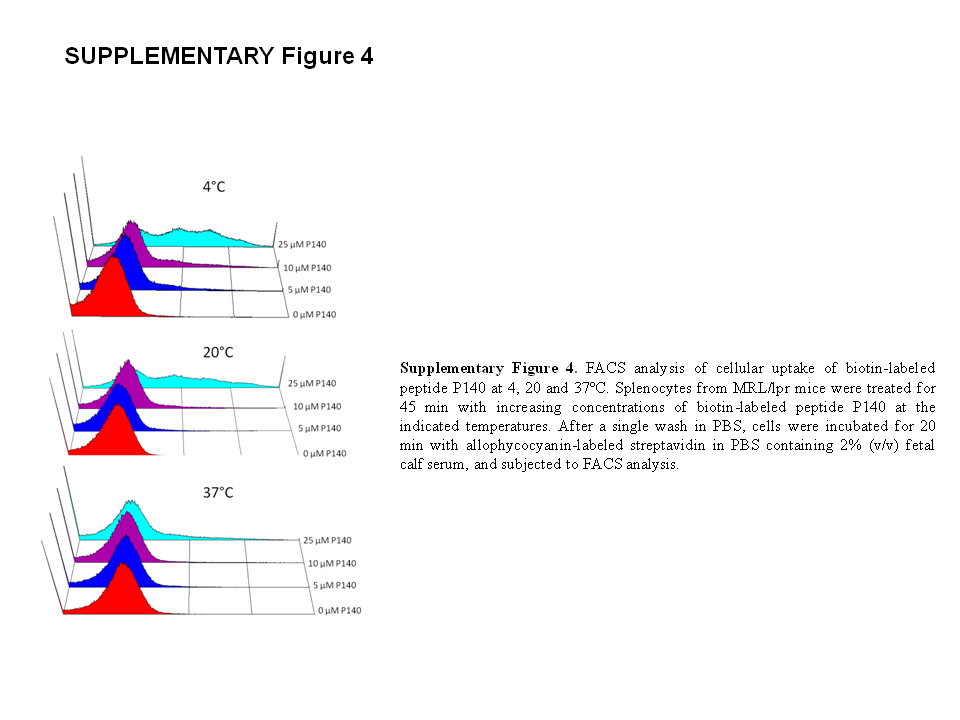

Supplement: Figure S4 — (0.12 MB TIF) [file pone.0005273.s004.tif]

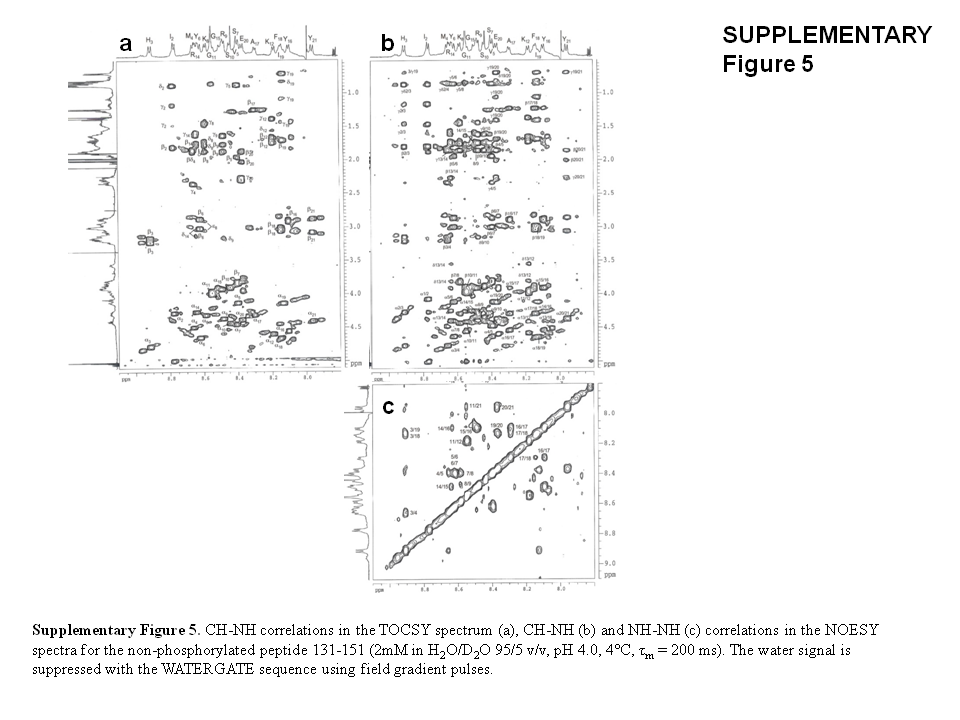

Supplement: Figure S5 — (0.24 MB TIF) [file pone.0005273.s005.tif]

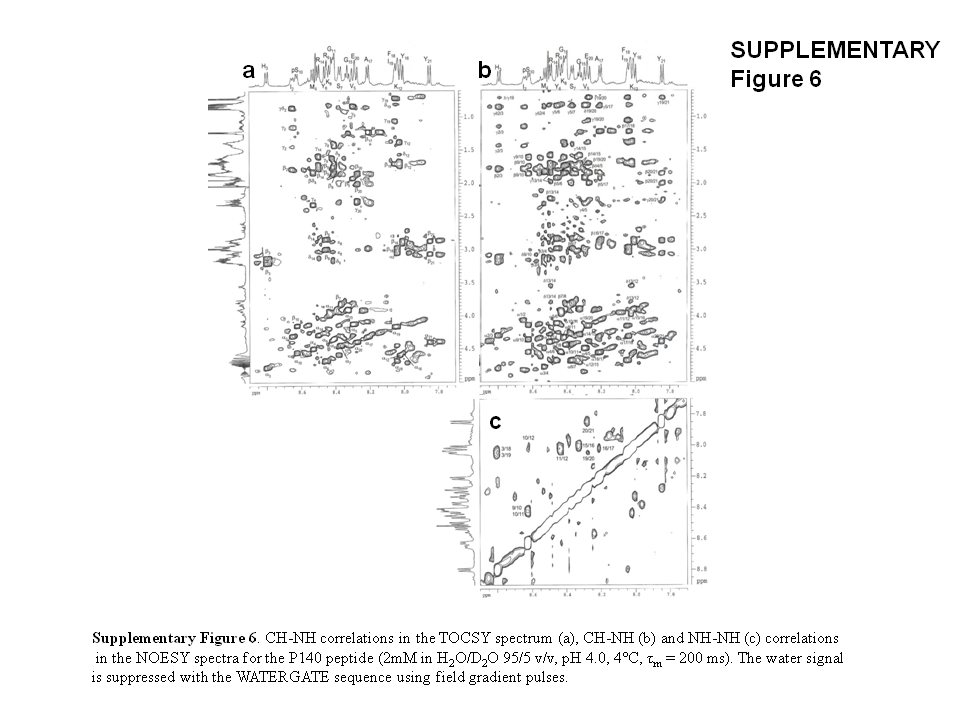

Supplement: Figure S6 — (0.24 MB TIF) [file pone.0005273.s006.tif]

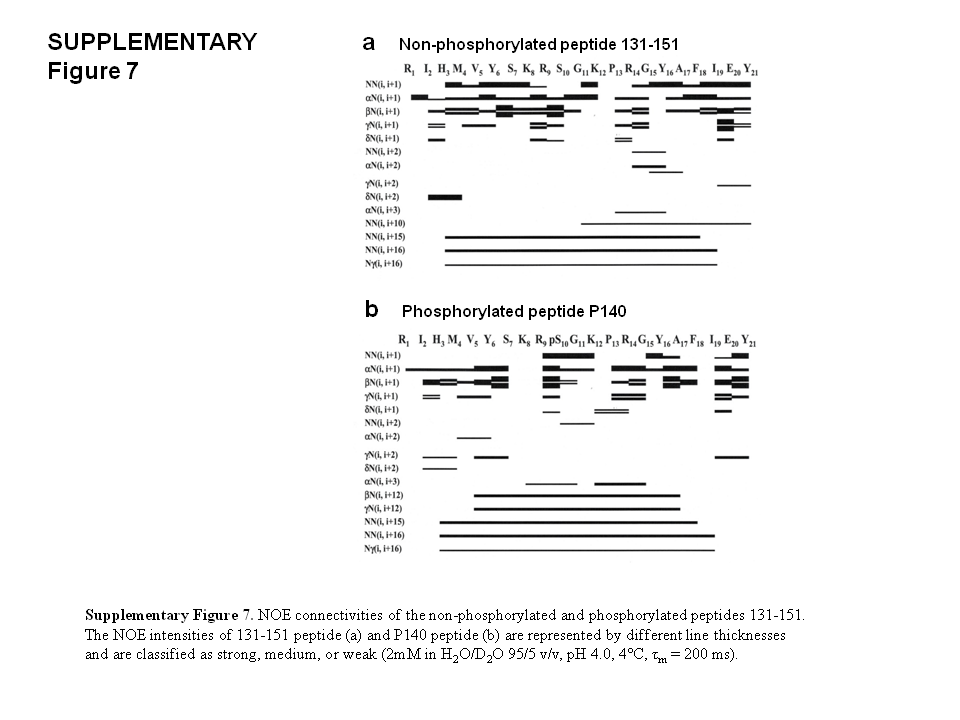

Supplement: Figure S7 — (0.16 MB TIF) [file pone.0005273.s007.tif]

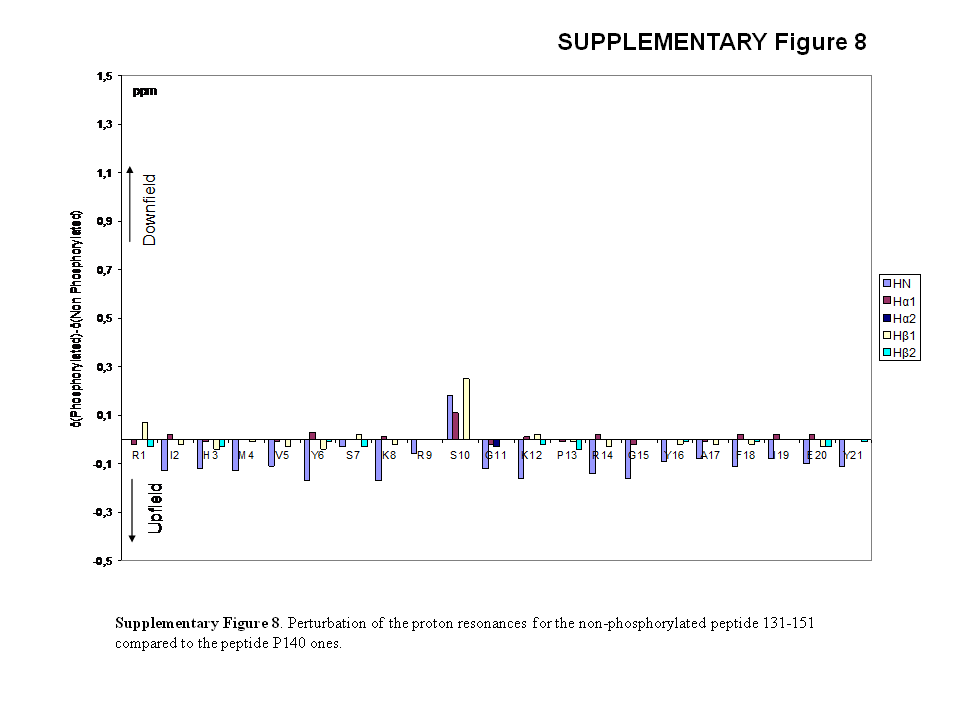

Supplement: Figure S8 — (0.07 MB TIF) [file pone.0005273.s008.tif]

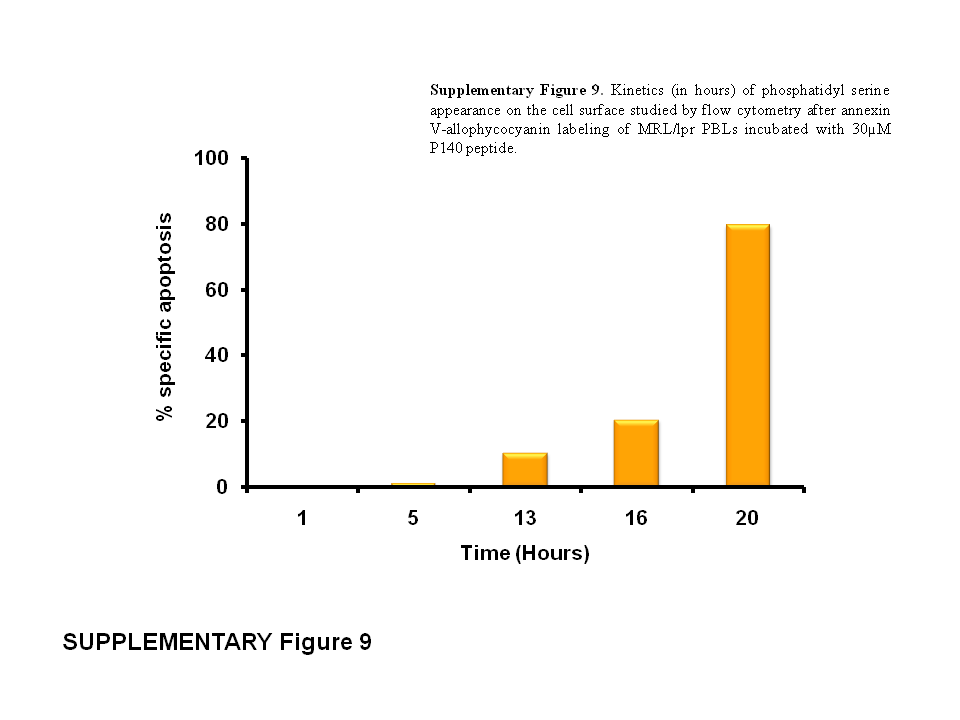

Supplement: Figure S9 — (0.08 MB TIF) [file pone.0005273.s009.tif]

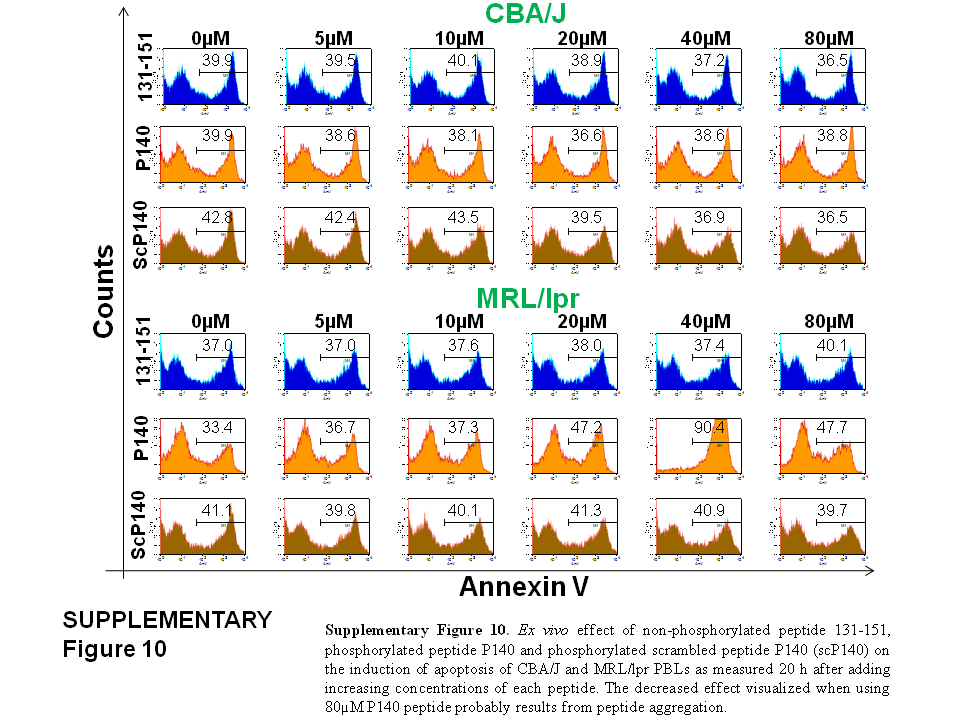

Supplement: Figure S10 — (0.19 MB TIF) [file pone.0005273.s010.tif]

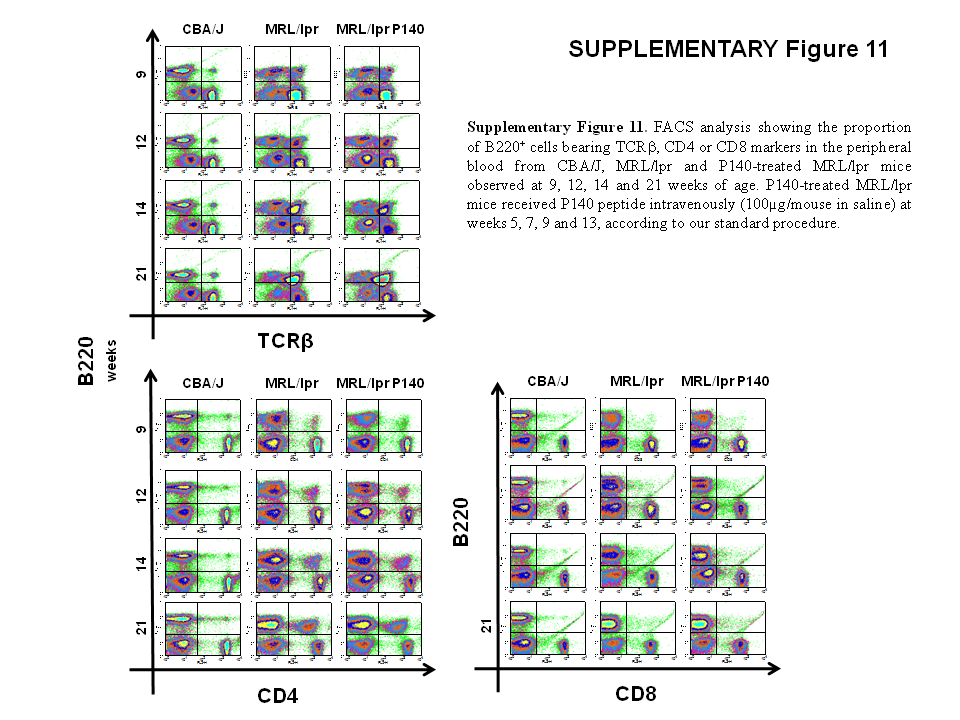

Supplement: Figure S11 — (0.43 MB TIF) [file pone.0005273.s011.tif]
