# Supplementary material for: The Spliceosomal Phosphopeptide P140 Controls the Lupus Disease by Interacting with the HSC70 Protein and via a Mechanism Mediated by γδ T Cells
Source: PLoS One. 2009 Apr 23;4(4):e5273. doi: 10.1371/journal.pone.0005273 (PMC2669294; doi:10.1371/journal.pone.0005273)
Supplement: Text S1 — Supporting Results (0.07 MB DOC) [file pone.0005273.s012.doc]

The spliceosomal phosphopeptide P140 controls the lupus disease by interacting with the HSC70 protein and *via* a mechanism

mediated by γδ T cells

Nicolas Page^1^, Nicolas Schall^1^, Jean-Marc Strub^2^, Marc Quinternet^3^, Olivier Chaloin^1^,

Marion Décossas^1^, Manh Thong Cung^3^, Alain Van Dorsselaer^2^, Jean-Paul Briand^1^

& Sylviane Muller^1^

^1^CNRS UPR9021, Institut de biologie moléculaire et cellulaire, Strasbourg, France

^2^CNRS UMR7178, laboratoire de spectrométrie de masse BioOrganique-IPHC-DSA- Université de Strasbourg, Strasbourg, France

^3^CNRS-INPL UMR7568, Laboratoire de Chimie-Physique Macromoléculaire, Nancy Université, ENSIC, Nancy, France

**Supplementary Results**

**NMR structure of phosphorylated (P140) and non-phosphorylated peptides 131-151 of the spliceosomal U1-70K protein**

**^1^H-NMR analysis*.*** For simplicity, in the figures and tables containing NMR data, peptide residues are numbered from 1 to 21 for the non-phosphorylated and phosphorylated peptides 131-151. The phosphorylated Ser(PO_3_H_2_) residue at position 10 (Ser^140^) is represented by pS. The sequence-specific resonance assignment was done according to the well-established procedure developed by Wüthrich [1] by using two-dimensional ^1^H, ^1^H TOCSY and NOESY experiments (Fig. S4 and S5). Sequential assignments of ^1^H resonances were based on characteristic sequential NOE connectivities between the proton α and the amide proton of residue *i* and the amide proton of residue *i+1*, *i.e.* αN(i, i+1) and NN(i, i+1) in the NOESY data set. The ^1^H chemical shifts are reported in Tables S1 and S2. Both peptides exhibited some negative NOE connectivities in aqueous solution, which denotes the presence of structured conformations. Fig. S6a and S6b summarize the sequential, medium and long range NOE connectivities for the non-phosphorylated and phosphorylated peptides, respectively. A 20/80 ratio of cis/trans conformers was observed on the TOCSY and NOESY of the non-phosphorylated and phosphorylated peptides for the K_12_-P_13_ amide bond due to the presence of P_13_ residue. The absence of any αα(K_12_, P_13_) NOE correlation denotes that the trans form is majority, the most populated.

The phosphorylation of the S_10_ residue leads to the observed slight upfield shifts of all of the NH protons excepted for that of the phosphorylated pS_10_ residue where downfield shifts are observed in addition to α and β protons (Fig. S7). Both peptides 131-151 and P140 present a similar NOESY spectral profile with numerous intraresidue and sequential NOE connectivities (Fig. S4b, S4c, S5b and S5c). However, the presence of strong NN(i, i+1) NOE cross-peaks, as a proof of the presence of helix or turn conformation [2,3], is observed only with NN(G_15_, Y_16_), NN(E_20_, Y_21_) NOE and in the R_9_pS_10_G_11_K_12_ segment of the phosphorylated peptide (Fig. S6b), while consecutive strong or medium NN(i, i+1) NOE cross-peaks are present in the H_3_M_4_V_5_Y_6_S_7_K_8_ and R_14_G_15_Y_16_A_17_F_18_I_19_E_20_Y_21_ segments of the non-phosphorylated peptide (Fig. S6a). For both peptides, no consecutive medium range (i, i+2) and (i, i+3) NOE connectivities are observed, suggesting the absence of any helix secondary structure. In the meantime, some NN(i, i+2), αN(i, i+2) and αN(i, i+3) cross-peaks were observed and diagnostic of a turn occurence. The presence of the weak long-range NN(G_11_, Y_21_) NOE cross-peak for non-phosphorylated peptide (Fig. S6a) and medium long-range βN(V_5_, A_17_) and γN(V_5_, A_17_) NOE cross-peaks for the phosphorylated peptide (Fig. S6b) at the same time with the presence of medium and weak NN(H_3_, F_18_), NN(H_3_, I_19_) and Nγ(H_3_, I_19_) long-range NOE connectivities for both peptides (Fig. S6) denote that both peptides adopt a β-hairpin structure.

**Structure calculations**. The ^3^J_αN_ coupling constants were obtained directly from the resolved amide proton resonances in the 1D spectra. The measured values of the ^3^J_αN_ coupling constants range from 5 to 9 Hz and correspond to the two sets of possible φ dihedral angle values around -80° and -160° for the L-amino acid residues [4]. Positive values of φ correspond to the disallowed regions in the Ramachandran plot. Except for those of Gly residues, the φ dihedral angles were constrained to be negative only within the -175° to -30° domain. For Gly residues, the ^3^J_αN_ coupling constants led to four possible values around ±70° and ±170°. The φ, ψ and χ_1_ values for the averaged structures, calculated from the chosen 50 lowest energy structures, are listed in Table S3. The more or less flexibility of the segments may be appreciated from the magnitude of the estimated standard deviations. The dimensions (H…O hydrogen bond distance and HNO angle) and the occurrence of the hydrogen bonds were determined by using the MOLMOL software and are given in Table S4.

Non-phosphorylated peptide*.* The peptide 131-151 exhibits several intense NN (i, i+1) NOE cross-peaks. The concomitant presence of the intense NN(G_15_, Y_16_) and NN(Y_16_, A_17_) NOE connectivities with the weak NN(R_14_, Y_16_) and weak or medium αN(R_14_, Y_16_) and αN(G_15_, A_17_) NOE connectivites (Fig. S6a) suggest the possible presence of a β-turn structure around the R_14_G_15_ and G_15_Y_16_ residues. The best fit for the superimposed peptide backbone gives a rmsd of 0.92±0.50 Å for the H_3_MVYSKRSGK_12_ segment (Fig. 2a) and a rmsd of 0.88±0.48 Å for the R_9_SGKPRGYAFIE_20_ segment. Although the φ dihedral angles of residues R_9_ (-69±15°), S_10_ (-77±10°), R_14_ (-92±1°), G_15_ (-67±3°), Y_16_ (-71±14°), A_17_ (-68±9°), I_19_ (-76±9°), and E_20_ (-84±8°) (Table S3), estimated by the MD simulation, are compatible with the presence of a distorted type I or type III β-turn, however due to the high ψ dihedral angle value of some residues, none of such a β-turn secondary structure with an occurrence higher than 10% was found. Instead of a β-turn, a γ-turn around the A_17_ residue (with a 88% occurrence of the F_18_-NH→Y_16_-CO hydrogen bonded interaction), and two π-turns (-57°, -70°) stabilized by the E_20_-NH®G_15_-CO (46% occurrence) and Y_21_-NH®Y_16_-CO (78% occurrence) intramolecular i+5®i H-bonds (Table S4) were identified. Two irregular loops were observed at the C-terminus whereas the N-terminus rather adopts an extended structure. A folded fragment was observed for the R_9_SGK_12_ sequence, which led to a final hairpin conformation as indicated by the presence of the long-range NOE connectivities. The S_10_ side-chain is directed towards the exterior medium, whereas the S_7_ side-chain is pointed at the inner part of the loop (Fig. 2a).

Phosphorylated peptide P140*.* Peptide P140 exhibits less NN(i, i+1) NOE correlations than the non-phosphorylated peptide. The presence of intense NN(R_9_, pS_10_), NN(pS_10_, G_11_) and NN(G_11_, K_12_) NOE correlations in addition to the weak or medium NN(pS_10_, K_12_), αN(K_8_, G_11_) and αN(K_12_, G_15_) NOE connectivities (Fig. S6b) are diagnostic of the presence of a folded structure in the K_8_RpSGKPRGY_16_ sequence.

The best fit for the superimposed peptide backbone gives a rmsd of 0.99 ± 0.62 Å for the S_7_KRSGKPRGY_16_ segment (Fig. 2b). One γ-turn, one β-turn and three α-turns were found. A γ-turn around the R_14_ residue is stabilized by the G_15_-NH → P_13_-CO (24% occurrence) hydrogen bond. The φ and ψ dihedral angles of residues R_14_ (-47°, -28°) and G_15_ (-60°, -5°) are compatible with the presence of a βI-turn stabilized by the Y_16_-NH®P_13_-CO (86% occurrence) hydrogen bond. The φ and ψ dihedral angles of residues K_8_ (-84°, -49°), R_9_ (-85°, -61°), pS_10_ (-60°, -40°) and G_11_ (-85°, -44°) are compatible with the presence of an α-turn (-63°, -42°) [5] stabilized by the K_12_-NH®K_8_-CO (68% occurrence) hydrogen bond. The two other α-turns are stabilized by the Y_16_-NH®K_12_-CO (10% occurrence) and Y_21_-NH®A_17_-CO (10% occurrence) hydrogen bonds, respectively (Table S4). The introduction of several long-range NOE correlations (Fig. S6b) in the MD calculation led to a hairpin structure. A loop is observed for the Y_6_SKRpSGK_12_ sequence, which contains the phosphorylated Ser residue. The phosphorylated side-chain of pS_10_ is also directed at the outside as also observed for the non-phosphorylated peptide. Thus, although the conformation of the two non-phosphorylated and phosphorylated peptides is slightly different, the side-chain of either the S_10_ or the pS_10_ residues is directed towards the exterior medium and is part of the hairpin loop, which probably plays a major role in the recognition of the phosphorylated pS_10_ residue by MHC molecules.

**Summary**. In the non-phosphorylated peptide 131-151 (Fig. 2a), two irregular loops are observed at the C-terminus whereas the N-terminus rather adopts an extended structure. A folded fragment is observed for the R^139^SGK^142^ sequence, which leads to a final hairpin conformation as indicated by the presence of the long-range NOE connectivities. The S^140^(S_10_) side-chain is directed towards the exterior medium, whereas the S^137^(S_7_) side-chain points at the inner part of the loop. P140 contains a folded structure in the K^138^RpSGKPRGY^146^ sequence (Fig. 2b). A loop is observed for the Y^136^SKRpSGK^142^ sequence, which contains the phosphorylated Ser residue (pS). The phosphorylated side-chain of pS^140^(pS_10_) is directed towards the outside, as also observed for the non-phosphorylated peptide. Thus, the conformation of the non-phosphorylated and phosphorylated peptides appears only slightly different. It should be emphasized in particular that in both peptide analogues, the side-chain of the Ser^140^ and pSer^140^ residues is directed towards the exterior medium and is part of the hairpin loop, which is probably important for recognition properties.

1. Wüthrich K (1986) NMR of Proteins and Nucleic Acids, Wiley, New York.
2. Sutcliffe MJ (1993) Structure determination from NMR data II. Computational approaches. NMR of macromolecules. A practical approach; Roberts, GCK, Ed., Oxford University Press, Oxford, pp 359-390.
3. Clore GM, Gronenborn, AM (1987) Determination of three-dimensional structures of proteins in solution by nuclear magnetic resonance spectroscopy. Prot Eng 1:275-288.
4. Cung MT, Marraud M, Néel J (1974) Experimental calibration of a Karplus relationship in order to study the conformations of peptides by Nuclear Magnetic Resonance. Macromolecules 7:606–613.
5. Toniolo C, Benedetti E (1991) The polypeptide 3_10_ helix. Trends Biochem Sci 16:350-353.
