# Supplementary material for: The Spliceosomal Phosphopeptide P140 Controls the Lupus Disease by Interacting with the HSC70 Protein and via a Mechanism Mediated by γδ T Cells
Source: PLoS One. 2009 Apr 23;4(4):e5273. doi: 10.1371/journal.pone.0005273 (PMC2669294; doi:10.1371/journal.pone.0005273)
Supplement: Table S1 — (0.03 MB DOC) [file pone.0005273.s013.doc]

**Supplementary Table S1**. Chemical shifts (ppm) of assigned proton resonances of the non-phosphorylated peptide 131-151

(RIHMVYSKRSGKPRGYAFIEY) in H_2_O/D_2_O (95/5 v/v)

Residue NH C^α^H C^β^H C^γ^H C^δ^H C^ε^H other

R_1_ 4.09 1.80, 1.90 1.57, 1.65 3.20 7.28 (N^η^H)

I_2_ 8.78 4.28 1.81 1.19, 1.48 0.89

Me 0.89

H_3_ 8.92 4.78 3.16, 3.23 7.25 8.57

M_4_ 8.66 4.50 1.84 2.35 1.89

V_5_ 8.40 4.13 1.95 0.88

Y_6_ 8.63 4.65 2.88, 3.01 7.06 6.76

S_7_ 8.40 4.45 3.81, 3.86

K_8_ 8.58 4.32 1.87 1.47 1.71, 1.77 3.01

R_9_ 8.48 4.36 1.76, 1.87 1.62, 1.65 3.18 7.26 (N^η^H)

S_10_ 8.44 4.44 3.90

G_11_ 8.55 3.95,4.00

K_12_ 8.18 4.59 1.65, 1.75 1.38 1.56 2.95

P_13_ 4.41 2.01, 2.28 1.87 3.57, 3.82

R_14_ 8.64 4.26 1.75, 1.78 1.60, 1.63 3.13 7.22 (N^η^H)

G_15_ 8.49 3.92

Y_16_ 8.10 4.55 2.88, 2.92 7.01 6.79

A_17_ 8.29 4.31 1.25

F_18_ 8.15 4.66 3.00, 3.06 7.34 7.2 7.28 (C^ς^H)

I_19_ 8.13 4.14 1.70 1.07, 1.30 0.82

Me 0.71

E_20_ 8.37 4.38 1.88, 2.03 2.15, 2.20

Y_21_ 7.96 4.40 2.88, 3.08 7.08 6.74
