# Supplementary material for: The Spliceosomal Phosphopeptide P140 Controls the Lupus Disease by Interacting with the HSC70 Protein and via a Mechanism Mediated by γδ T Cells
Source: PLoS One. 2009 Apr 23;4(4):e5273. doi: 10.1371/journal.pone.0005273 (PMC2669294; doi:10.1371/journal.pone.0005273)
Supplement: Table S2 — (0.03 MB DOC) [file pone.0005273.s014.doc]

**Supplementary Table S2**. Chemical shifts (ppm) of assigned proton resonances of the phosphorylated peptide P140

(RIHMVYSKRpSGKPRGYAFIEY) in H_2_O/D_2_O (95/5 v/v)

Residue NH C^α^H C^β^H C^γ^H C^δ^H C^ε^H other

R_1_ 4.07 1.87 1.58, 1.64 3.18 7.18 (N^η^H)

I_2_ 8.65 4.30 1.79 1.15, 1.45 0.85

Me 0.85

H_3_ 8.80 4.77 3.12, 3.20 7.21 8.51

M_4_ 8.53 4.50 1.83 2.33 1.83

V_5_ 8.29 4.12 1.92 0.85

Y_6_ 8.46 4.68 2.84, 3.00 7.05 6.76

S_7_ 8.37 4.45 3.83

K_8_ 8.41 4.33 1.85 1.41, 1.45 1.68 2.98

R_9_ 8.42 4.36 1.76, 1.87 1.61, 1.65 3.16 7.24 (N^η^H)

pS_10_ 8.62 4.55 4.15

G_11_ 8.43 3.93, 3.97

K_12_ 8.02 4.60 1.67, 1.73 1.38 1.58 2.94

P_13_ 4.40 2.00, 2.24 1.87 3.55, 3.80

R_14_ 8.50 4.28 1.72, 1.78 1.59, 1.63 3.12 7.15 (N^η^H)

G_15_ 8.33 3.90

Y_16_ 8.01 4.55 2.86, 2.91 7.01 6.79

A_17_ 8.21 4.30 1.23

F_18_ 8.04 4.68 2.98, 3.05 7.31 7.18 7.25 (C^ς^H)

I_19_ 8.05 4.16 1.70 1.07, 1.30 0.80

Me 0.71

E_20_ 8.27 4.40 1.85, 2.00 2.27

Y_21_ 7.85 4.40 2.88, 3.07 7.07 6.73
