# Supplementary material for: The Spliceosomal Phosphopeptide P140 Controls the Lupus Disease by Interacting with the HSC70 Protein and via a Mechanism Mediated by γδ T Cells
Source: PLoS One. 2009 Apr 23;4(4):e5273. doi: 10.1371/journal.pone.0005273 (PMC2669294; doi:10.1371/journal.pone.0005273)
Supplement: Table S3 — (0.04 MB DOC) [file pone.0005273.s015.doc]

**Supplementary Table S3**. Averaged φ, ψ, and χ_1_ values (degrees) of the final set of 50 structures for the non-phosphorylated and phosphorylated peptide 131-151 (RIHMVYSKRSGKPRGYAFIEY)

Residue Torsion angle Non-phosphorylated Phosphorylated

φ_NMR_ MD simulation φ_NMR_ MD simulation

R_1_ ψ 111 ± 17 113 ± 87

χ_1_ -157 ± 70 168 ± 71

I_2_ φ -72; -168 -125 ± 25 -73; -167 -117 ± 24

ψ 55 ± 6 153 ± 17

χ_1_ 150 ± 6 57 ± 18

H_3_ φ -77; -163 -80 ± 4 -78; -162 -92 ± 8

ψ -62 ± 5 -70 ± 5

χ_1_ -158 ± 18 -168 ± 25

M_4_ φ -73; -167 -145 ± 13 -74; -166 -138 ± 12

ψ -109 ± 21 94 ± 14

χ_1_ -136 ± 59 -161 ± 66

V_5_ φ -79; -161 -172 ± 17 -82; -158 -94 ± 21

ψ 93 ± 10 145 ± 5

χ_1_ -85 ± 36 -67 ± 5

Y_6_ φ -74; -166 -97 ± 12 -77; -163 -69 ± 14

ψ -69 ± 5 -90 ± 17

χ_1_ -88 ± 6 -63 ± 5

S_7_ φ -71; -169 -147 ± 17 -75; -165 -122 ± 25

ψ -64 ± 3 -157 ± 42

χ_1_ -79 ± 49 -156 ± 87

K_8_ φ -69; -171 -129 ± 20 -71; -169 -84 ± 10

ψ 175 ± 9 -49 ± 11

χ_1_ 170 ± 47 -131 ± 94

R_9_ φ -71; -169 -69 ± 15 -72; -168 -85 ± 7

ψ -76 ± 8 -61 ± 1

χ_1_ -82 ± 8 -89 ± 8

S_10_/p S_10_ φ -72; -168 -77 ± 10 -72; -168 -60 ± 11

ψ 113 ± 9 -40 ± 5

χ_1_ -166 ± 84 102 ± 84

G_11_ φ -148 ± 15 -85 ± 4

ψ 62 ± 7 -44 ± 20

K_12_ φ -72; -168 -16 ± 17 -79; -161 -116 ± 15

ψ -56 ± 9 64 ± 37

χ_1_ -108 ± 50 -13 ± 88

P_13_ ψ -60 ± 5 -106 ± 33

R_14_ φ -69; -171 -92 ± 1 -71; -169 -47 ± 9

ψ -57 ± 4 -28 ± 6

χ_1_ 73 ± 22 71 ± 17

G_15_ φ -67 ± 3 -60 ± 9

ψ 1 ± 4 -5 ± 11

Y_16_ φ -71; -169 -71 ± 14 -75; -165 -50 ± 4

ψ -45 ± 9 -50 ± 2

χ_1_ -168 ± 7 -103 ± 4

A_17_ φ -71; -169 -68 ± 9 -73; -167 -103 ± 18

ψ 3 ± 1 97 ± 9

χ_1_ -3 ± 109 144 ± 102

F_18_ φ -77; -163 -167 ± 8 -77; -163 -81 ± 13

ψ -16 ± 8 -21 ± 11

χ_1_ -102 ± 8 -150 ± 16

I_19_ φ -75; -165 -76 ± 9 -82; -158 -52 ± 4

ψ -61 ± 2 -51 ± 2

χ_1_ -42 ± 11 -12 ± 7

E_20_ φ -76; -164 -84 ± 8 -78; -162 -68 ± 11

ψ -41 ± 1 -61 ± 1

χ_1_ -154 ± 28 -97 ± 3

Y_21_ φ -79; -161 -108 ± 30 -78; -162 -102 ± 17

χ_1_ -162 ± 50 -91 ± 74
