# Supplementary material for: The Spliceosomal Phosphopeptide P140 Controls the Lupus Disease by Interacting with the HSC70 Protein and via a Mechanism Mediated by γδ T Cells
Source: PLoS One. 2009 Apr 23;4(4):e5273. doi: 10.1371/journal.pone.0005273 (PMC2669294; doi:10.1371/journal.pone.0005273)
Supplement: Table S4 — (0.03 MB DOC) [file pone.0005273.s016.doc]

**Supplementary Table S4**. Dimension (Å, degrees) and occurrence (%) of the hydrogen bonds in the non-phosphorylated and phosphorylated peptides estimated with the 50 lowest energy chosen structures

HB dimensions

Hydrogen distance angle

Donor Acceptor bond type turn H···O <HNO Occurrence

Non-phosphorylated peptide 131-151

H_3_-HN F_18_-CO 2.1 - 2.4 9 - 50 10

Y_16_-HN R_14_-CO *i* + 2→*i* γ 2.4 - 2.5 30 - 39 10

F_18_-HN Y_16_-CO *i* + 2→*i* γ 1.9 - 2.5 10 - 27 88

I_19_-HN G_15_-CO *i* - 4→*i* α 2.3 - 2.4 34 - 46 10

E_20_-HN G_15_-CO *i* + 5→*i* π 1.9 - 2.4 3 - 27 46

E_20_-HN Y_16_-CO *i* + 4→*i* α 2.4 - 2.5 20 - 31 10

Y_21_-HN Y_16_-CO *i* + 5→*i* π 2.0 - 2.5 18 - 47 78

Phosphorylated peptide P140

K_12_-HN K_8_-CO *i* + 4→*i* α 2.1 - 2.5 24 - 37 68

G_15_-HN P_13_-CO *i* + 2→*i* γ 2.4 - 2.5 35 - 39 24

Y_16_-HN K_12_-CO *i* + 4→*i* α 2.2 - 2.5 39 - 55 10

Y_16_-HN P_13_-CO *i* + 3→*i* βIII 2.0 - 2.5 12 - 33 86

Y_21_-HN A_17_-CO *i* + 4→*i* α 2.0 - 2.5 15 - 33 46
